# Numerical study of the tire hydroplaning behavior of aircraft on grooved concrete pavement

**DOI:** 10.1371/journal.pone.0292701

**Published:** 2023-11-01

**Authors:** Jing Cai, Nizhi Du, Ning Zhou, Yue Li, Xuan Dai, Heng Zhang

**Affiliations:** 1 College of Transportation Science and Engineering, Civil Aviation University of China, Tianjin, China; 2 Engineering Research Center of Intelligent Construction and Industrialization, Civil Aviation Administration of China, Beijing, China; 3 Airport Management Institute, Shanghai Civil Aviation College, Shanghai, China; Shandong University of Technology, CHINA

## Abstract

Safe operation is crucial for civil aviation, and reducing the risk of aircraft tire hydroplaning is essential for civil aviation safety. Here, a new 3D aircraft tire-grooved (smooth) wet pavement model based on the coupled Eulerian-Lagrangian (CEL) algorithm for the A320 aircraft was developed, and the effect of the ground contact area of an aircraft tire on the hydrodynamic pressure and support force of the tire under smooth and grooved wet pavement conditions was investigated. The results indicate that at the same taxiing speed, the ground contact area of the aircraft tire under the grooved wet-pavement condition is reduced by 19.8% compared to the smoothed wet-pavement condition, which is reduced by 6.2%. Similar patterns are observed for the hydrodynamic pressure and the critical hydrodynamic speed during landing and taking-off procedures, with upper and lower limited values obtained through the simulation results. Additionally, the predicted correction factor of the hydroplaning speed at different water film thicknesses is compared with those values obtained via the NASA formula. A comparison shows that the NASA formula underestimates the critical hydroplaning speed during the landing procedure. The corresponding correction factor will be less than 1.0 when the water film thickness reaches a critical value of 7.66 mm.

## Introduction

Tire hydroplaning refers to a special wheel skidding phenomenon that occurs when an aircraft is taxiing on wet pavement, which is one of the most common factors affecting the safe operation of civil aviation aircraft [1]. An aircraft may slide off the runway and crape into the green belt under wet-pavement conditions, leading to an aviation accident. Factors such as aircraft type, water film thickness, and pavement condition may contribute to tire hydroplaning. Related mechanisms should be studied to improve the performance of civil aviation operations.

First, the National Aeronautics and Space Administration (NASA) carried out aircraft tire hydroplaning tests. On the basis of an extensive amount of data, the critical hydroplaning speed formula, which is known as the NASA formula, was proposed [2]. The critical hydroplaning speed involved in this formula is crucial for studying tire hydroplaning behavior. Additionally, tire hydroplaning experiments were conducted to investigate the effects of factors such as pavement roughness, tire tread depth, tire pressure, and load on tire hydroplaning performance [3]. Due to the high test cost and strict test conditions, the study of tire hydroplaning is mainly carried out by theoretical calculation and simulation analysis.

The theoretical calculation usually involves constructing a theoretical model of the critical hydroplaning speed and predicting and verifying the critical hydroplaning speed by referring to various types of aircraft and corresponding tire models [4]. Theoretical calculations show that two factors, water film thickness and tire pressure, have a great influence on the critical speed of water skiing [5]. Compared with the tire hydroplaning test, the theoretical calculation is simpler. However, it is limited by its complex boundary conditions and some disparities with the real situation. In contrast, the use of numerical simulations of tire hydroplaning phenomena is currently the mainstream method. The Computational Fluid Dynamics (CFD) method and coupled Eulerian-Lagrangian (CEL) method are the most widely used. The CFD method combined with the FEM method can be used to study the critical hydroplaning speed of aircraft tires, automobile tires and driving resistance [6]. Likewise, the CEL method can better avoid the mesh distortion caused by the Lagrangian method when analyzing large deformations. When the hydroplaning problem of tires with complex treads is studied, the water film and tire interaction mechanism is further revealed [7–9]. Other studies have shown that the force on tires is different when a vehicle is hydroplaning due to the direction, location and area of the grooves on the pavement compared to smooth pavement [10]. CEL numerical simulation results also show that the critical hydroplaning speed on a wet grooved pavement is reduced compared to a smooth pavement [11]. In addition, increasing the thickness of the water film can also reduce the speed [12].

To summarize, the existing way to study the hydroplaning problem of aircraft tires is to simulate the numerical analysis software. In addition, the above studies on tire hydroplaning speed are all based on smooth pavement or low tire pressure, low axle load of aircraft or automobile tires. More than 90% of the existing airport pavement in China is cement concrete pavement. To achieve the friction requirements, the pavement needs to be grooved. Moreover, the high tire pressure and high axle load of large aircraft has become the mainstream of civil aviation [13]. Unlike previous studies of critical hydroplaning speed based on low tire pressure, low axle load aircraft tires, or automobile tires used to guide aircraft takeoffs and landings on wet pavement, the safety of these studies is difficult to guarantee.To study the effect of water film thickness and pavement condition on tire hydroplaning behavior, in this paper, the CEL algorithm is adopted to perform a series of numerical simulations for the A320 aircraft via the commercial finite element software ABAQUS. The critical hydroplaning speed during aircraft landing is obtained by analyzing the interaction mechanism between tires and smooth pavement and tires and textured pavement with this model. Further analysis is performed regarding the influence of pavement conditions and aircraft type on the critical hydroplaning speed under wet and grooved pavement states. The parameters of the NASA formula are modified.

## Materials and methods

The tire-wet pavement interaction model consists of a solid tire and textured pavement, which are both solid modules, and a water film, which is a liquid module. Therefore, CEL fluid-structure coupling algorithm was adopted and 7.66mm was selected as the water film thickness according to Horne test.

### Solid tire model

**Fig 1:**
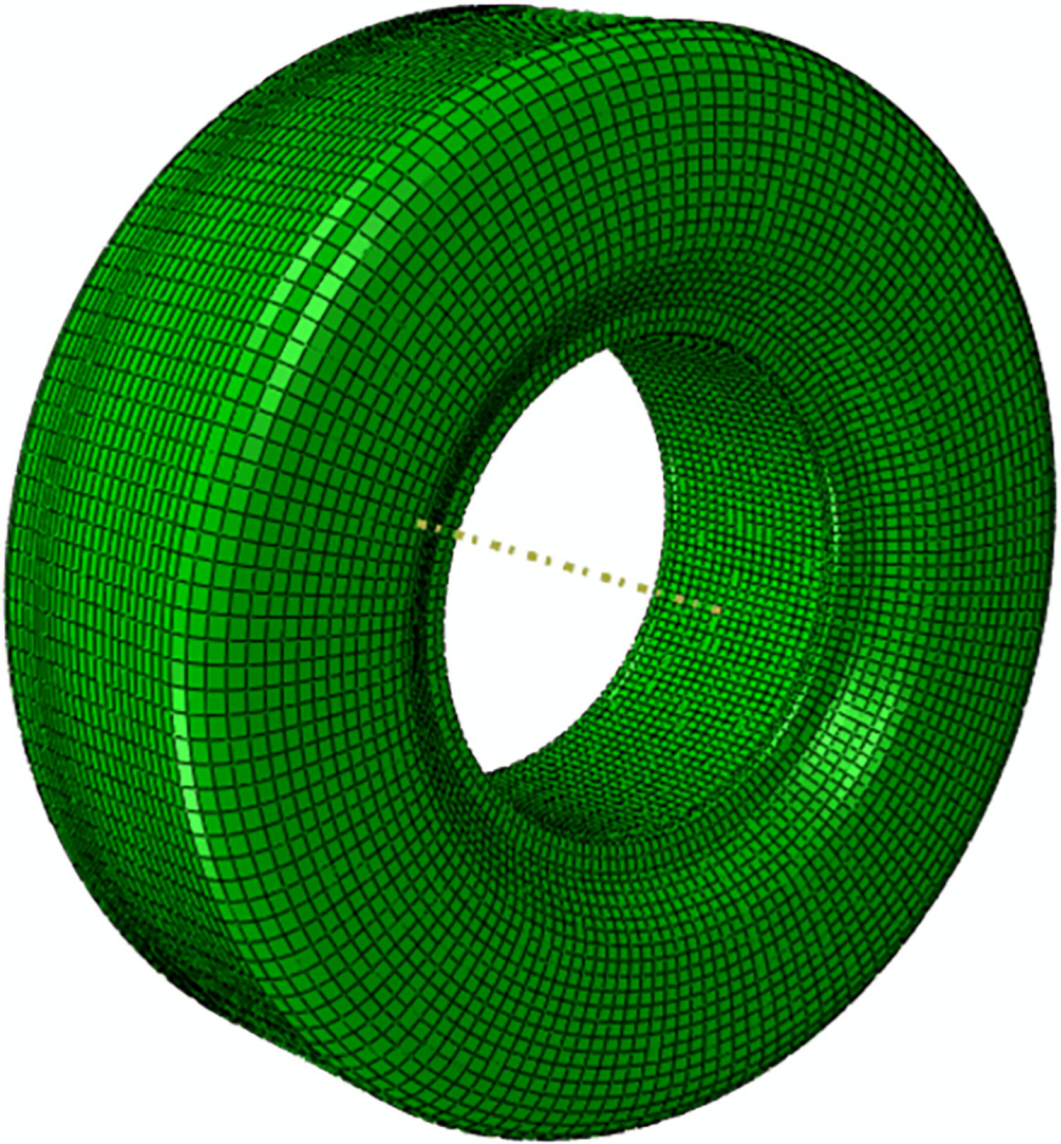
A320 tire model.

In this paper, the A320 type 46 × 17R20 main undercarriage tire with load distribution of more than 90% are selected. The model mesh is evenly divided into sections with the tire diameter center as the turning center and evenly divided by angles, and the total number of units is 40996 (Fig 1). It is assumed that the inner cord and the tire surface outer layer are hyperelastic homogeneous rubber materials [14] (Table 1).

**Table 1:** Parameters of the aircraft tire on the main gear.

Parameter	Value
Tire diameter *D*/cm	115
Tire width *B*/cm	43
Tire pressure *P*/kPa	1140
Rubber positive constant C_10_	9.9×10^6^
Rubber positive constant *C*_01_	8.8×10^6^
Rubber incompressibility constant *D*_1_	10^−7^
Single wheel axle load *W*/kN	152

### Hydrops pavement model

**Fig 2:**
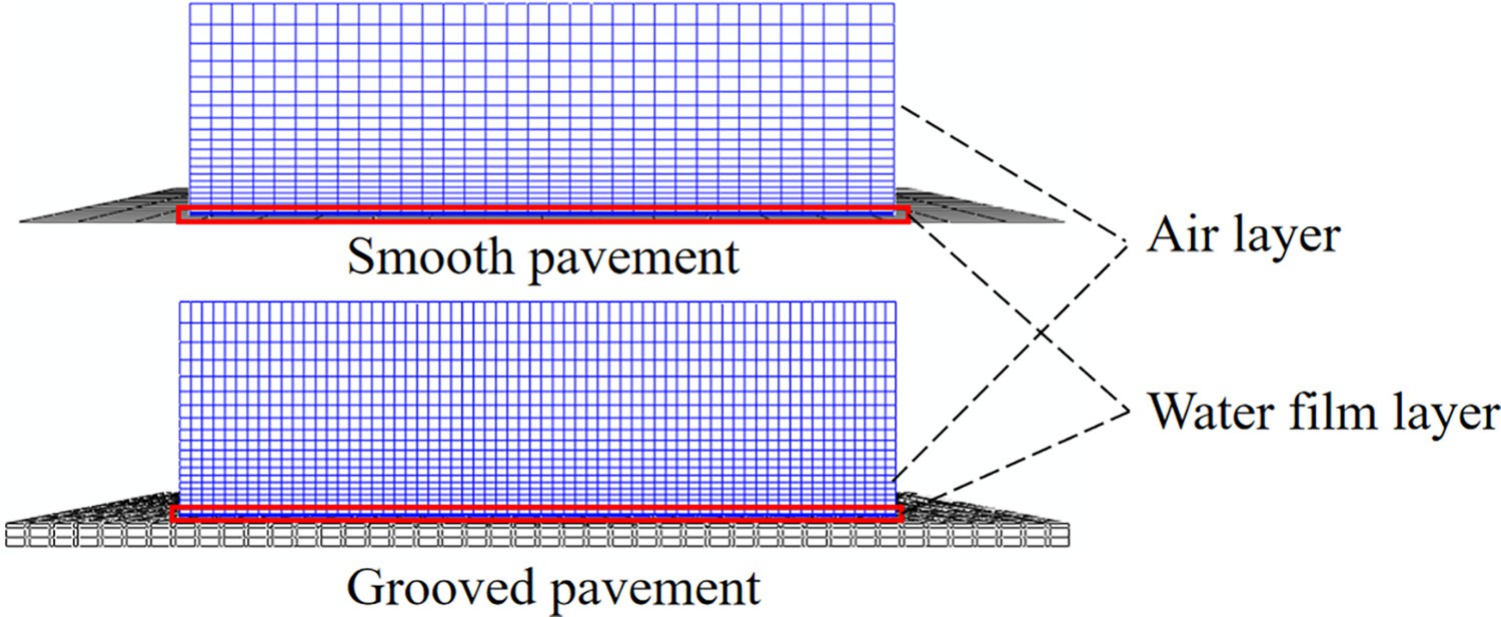
Wet pavement model.

Here, a water film is used to explore and compare the interaction of a tire with a smooth pavement and a grooved pavement during hydroplaning [15, 16]. The width and depth of the grooves are 6 mm, and the longitudinal spacing is 32 mm (Fig 2).

To characterize the liquid flow, the fluid layer’s total thickness is set to 300 mm, the water film thickness is set to 7.66 mm, and the remainder is the air layer. The pavement model size is 1.4 m × 1.4 m, and the fluid plane is 1 m × 1 m. In order to realistically simulate the flow characteristics of the liquid when the tire drives through a water film, the front edge of the model is set as the inlet, the rear edge is set as the water outlet, and the bottom and both sides are set as the impermeable boundaries (Fig 2). The grid is identified step by step from the top to the bottom to ensure the calculation accuracy of critical contact surfaces during the calculation and optimize the calculation efficiency. The number of water film units is 75900, the smooth pavement units are 361, and the grooved pavement units are 3402. Table 2 presents the parameters of the fluid layer, with dynamic viscosity serving as a characterization of its viscosity. The fluid pressure and density changes are solved through Mie-Grüneisen Equation, expressed as follows:

$$\begin{array}{l} p=f(\rho )+g(\rho )E_{m}\\ f(\rho )=\rho_{w}^{0}c_{0}^{2}\gamma (1-\Gamma_{0}\gamma /2)/[(1-s\gamma )]\\ g(\rho )=\Gamma_{0}\rho_{w}^{0} \end{array}$$ (1)

where *p* is the dynamic water pressure, *ρ* is the fluid density, $\rho_{w}^{0}$ is the initial density of water, *c*_0_ is the propagation velocity of a sound wave in a fluid, *s* and *γ* are the nominal volume compression strain, Γ_0_ is a dimensionless material constant, *E*_*m*_ is the specific internal energy.

**Table 2:** Water film parameters.

$\rho_{w}^{0}$ kg/m^3^	Mie-Grüneisen Parameters				*n*_*w*_ N·s/m^2^	*E*_*m*_ J/kg
	*c*_0_ m/s	*γ*	*s*	Γ_0_		
1000	1480	0.11	1.979	0.11	1.0×10^3^	0

*n*_*w*_ is the dynamic viscosity of water representing the internal friction coefficient of the liquid.

### Three-dimensional tire-wet pavement interaction model

**Fig 3:**
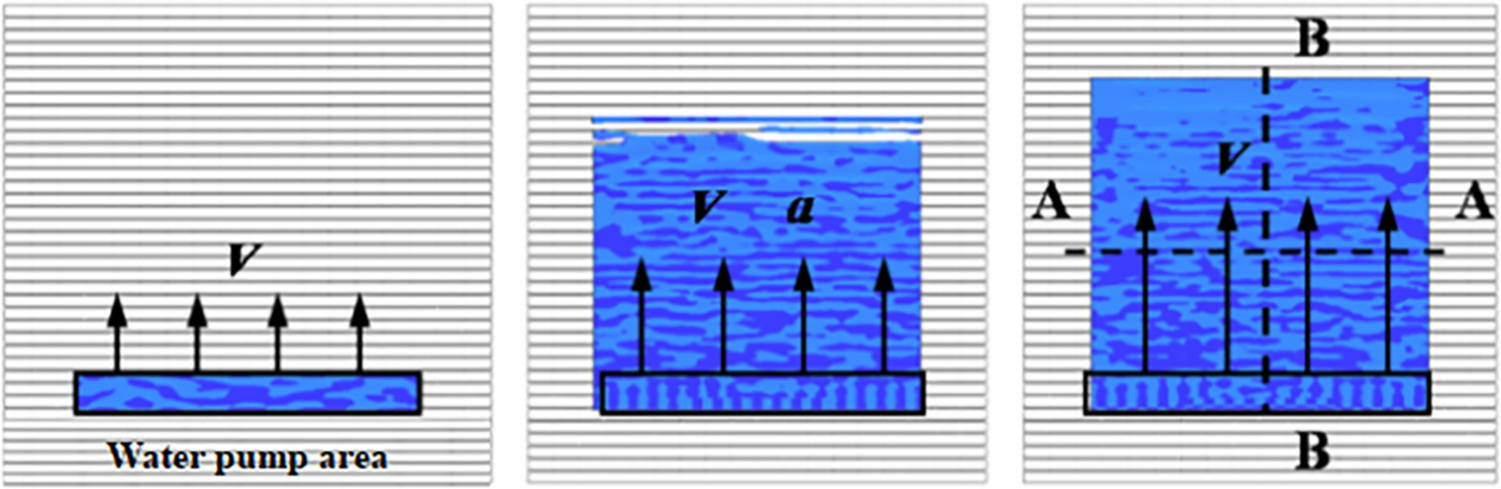
Wet pavement forming process.

**Fig 4:**
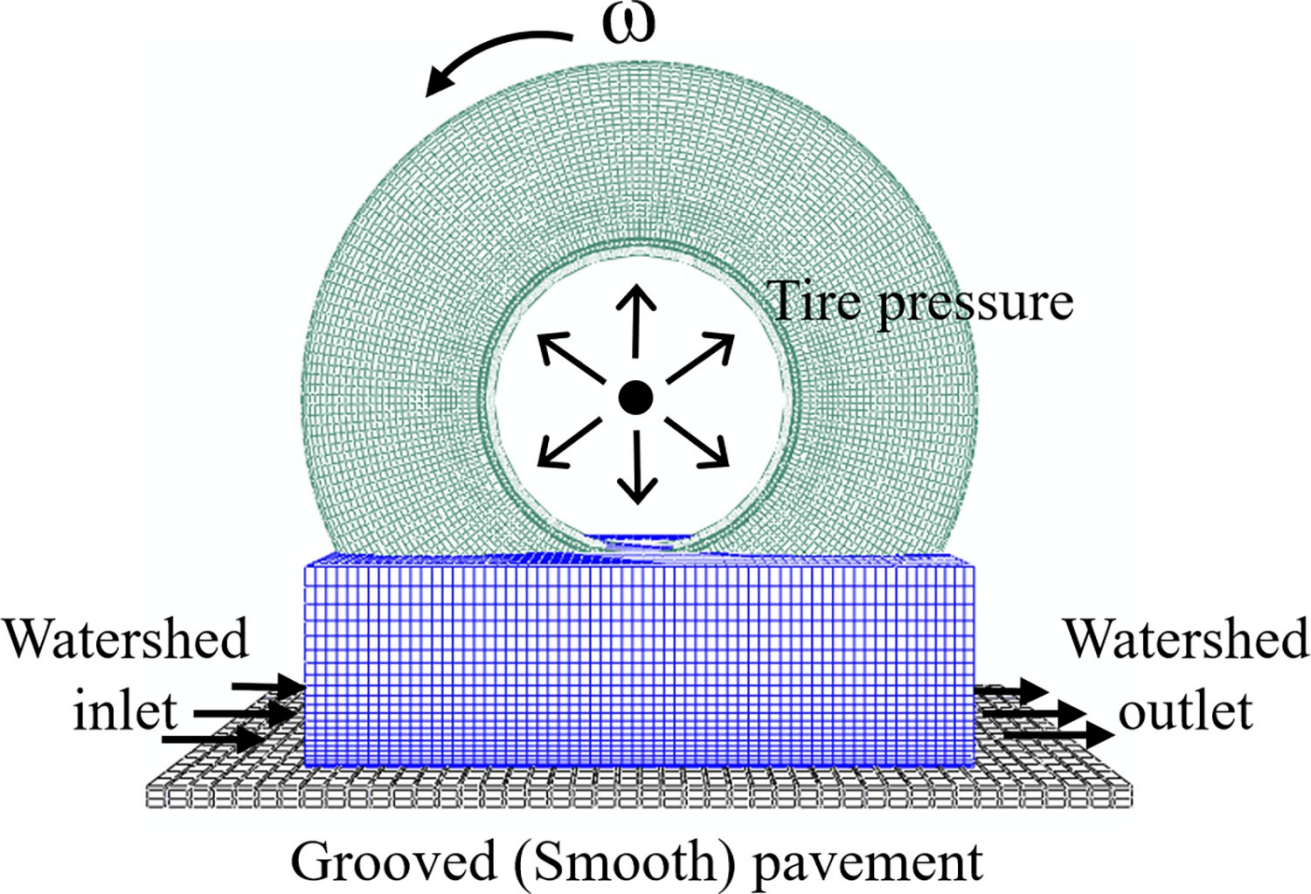
3D model of tire-wet pavement interaction.

To characterize the interaction between the tire and wet pavement and optimize the calculation efficiency, the tire is rotated around the wheel center O at a palstance *ω*. A water film impacts the tire, and initial speed *v* and acceleration a are set in the pump area to maintain the stability of the watershed under a constant impact speed. The specific process of the formation of wet pavement is shown in Fig 3, and the uniform tire pressure *P* and wheel load *F* are applied to the tire’s inner wall [17, 18]. The rotating tire and the wet pavement formed a three-dimensional tire-wet pavement interaction model (Fig 4).

### Tire model verification

**Fig 5:**
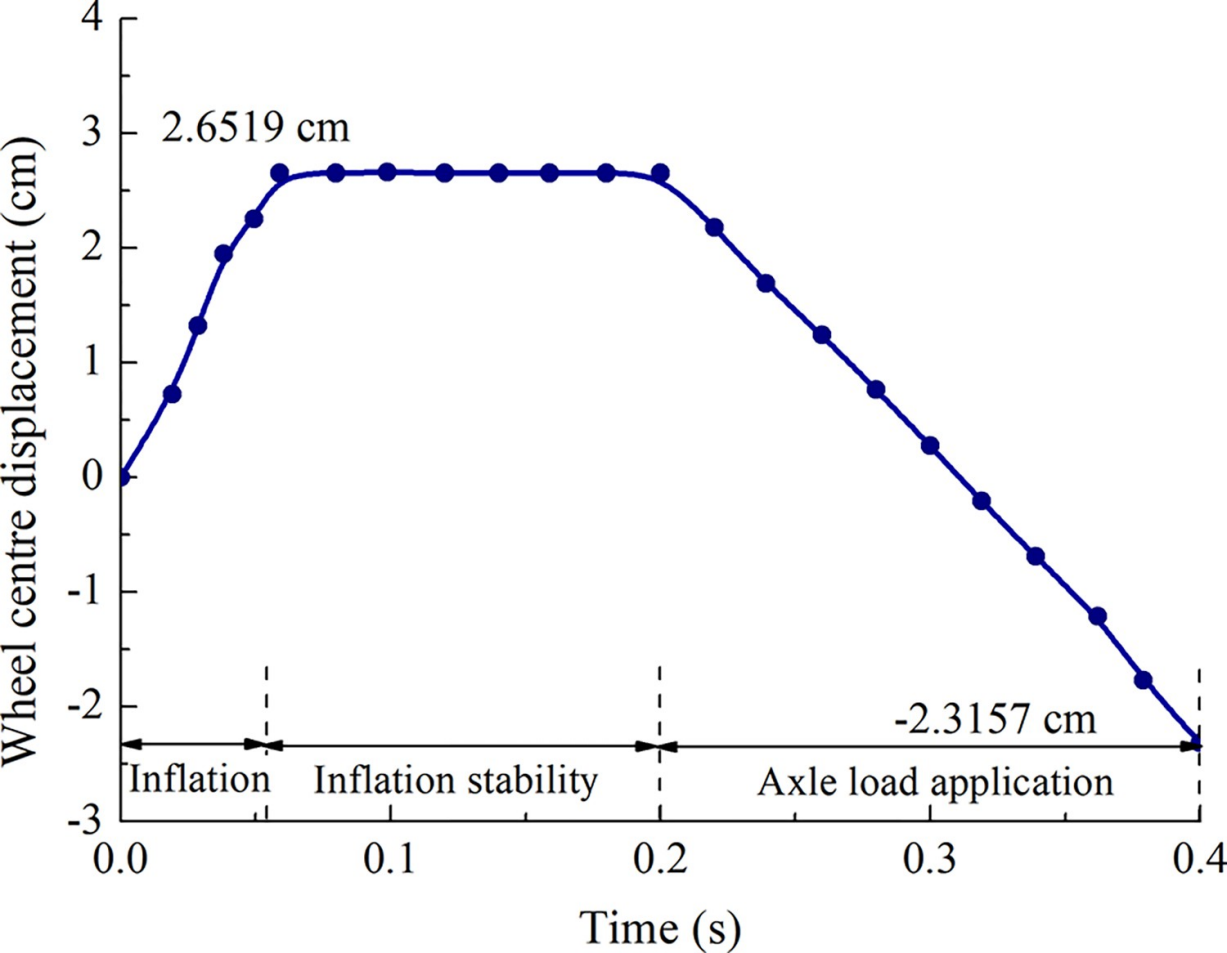
Vertical displacement of the tire rotation center.

The vertical stiffness of the tire in the simulation process is mainly related to the section size and material parameters that affect the tire’s vertical displacement [19, 20]. The change in vertical displacement is primarily in the actual inflation and pressurization stage, which includes inflation (exert tire pressure), a stabilization period after inflation, and application of the sheer concentrated force of the main landing gear (exert axle load). The displacement change in the tire center point during the process is shown in Fig 5.

The vertical displacement is increased to 2.652 cm in the inflation stage, and the tire center point subsidence is 4.968 cm in the axle load application stage. Taking the A320 parameters into the vertical tire displacement empirical formula [21]. The calculation formula is as follows:

$$\Delta =\frac{kQ_{d}^{0.85}}{B^{0.7}D^{0.43}P^{0.6}}\left(15\times 10^{-3}B+0.42\right)$$ (2)

Where Δ is the amount of compression deformation of the tire (cm), *k* is the parameter related to tire design, typically taken as 1.5 for radial tires, *Q*_*d*_ is the load on the tire (N), *B* is the tire width (cm), *D* is the tire outside diameter (cm), and *P* is the internal pressure of the tire (kPa).

The calculation result is 4.968 cm. The relative difference between the results of the calculation and the model simulation is 6.4% considering that the vertical stiffness of the tire model is reasonable.

### Verification of the fluid field stability and rationality

**Fig 6:**
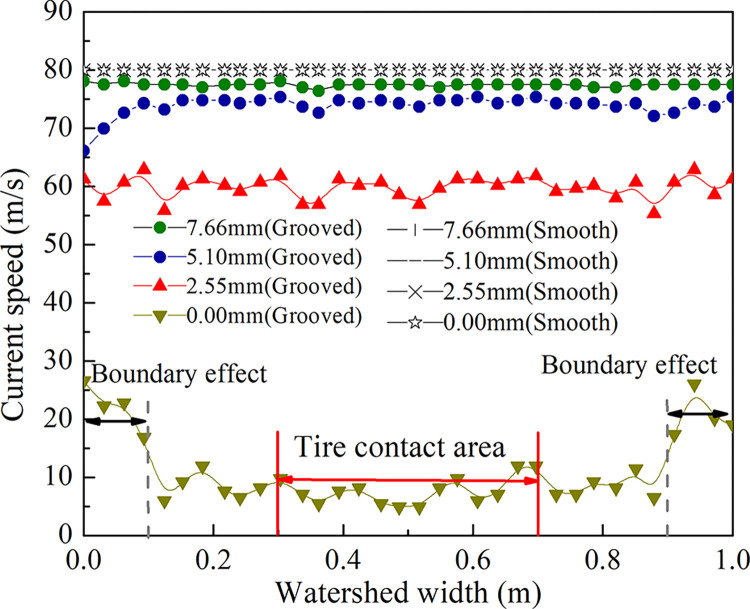
The flow velocities of the different water layers in section A-A.

**Fig 7:**
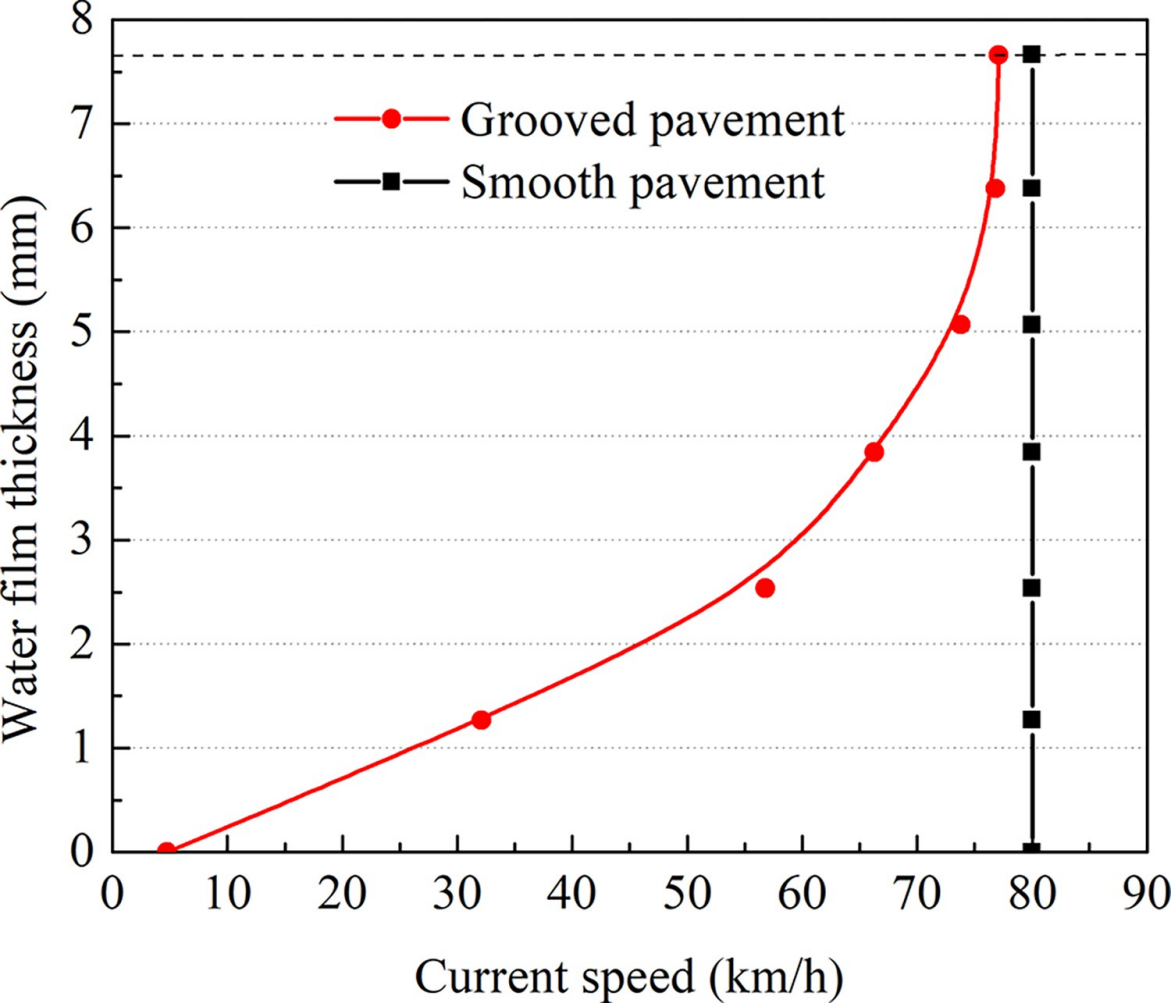
The flow velocity of the fluid field center.

According to the different pavement types given, to verify the rationality of the size selection, the initial velocity of the fluid is 80 m/s. The flow velocity distribution of the water film on the grooved and smooth pavements at the intersection of sections A-A and the watershed center (the section intersection of A-A and B-B) in Fig 3 is extracted, and the results are shown in Figs 6 and 7.

Fig 7 shows that the flow velocity at each depth on the smooth pavement is 80 m/s, the velocity of the water film changes in a curve along the depth direction, the speed at the bottom layer is 5 m/s, and the speed at the upper layer is 80 m/s. These results indicate the blocking effect of the pavement texture on water flow and the ponding model’s rationality. Considering the boundary effect in the tire action area, the boundary on both sides of the smooth pavement does not affect the flow velocity, while the grooved pavement influence width is within 0.1 m (Fig 6). Therefore, the selection of a 1 m watershed can eliminate the influence of the boundary effect.

**Fig 8:**
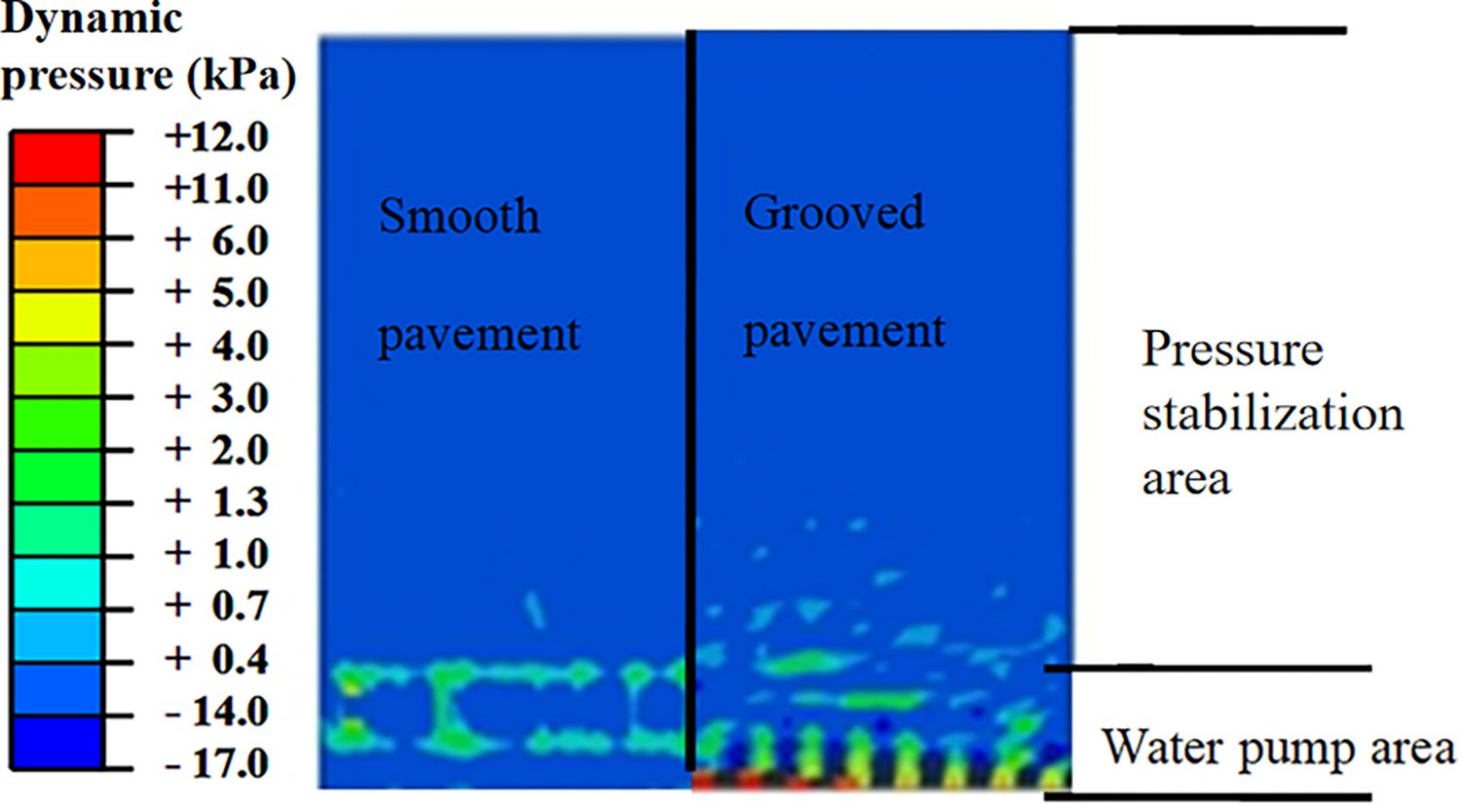
Diagram of the hydrodynamic pressure on the water surface.

Fig 8 shows that the pressure in the water pump area of the initial flow field of the smooth pavement is high and that the pressure distribution in other areas is stable [22–24]. The influence area of the water pump area of the grooved pavement is more significant than that of the smooth pavement, while the pressure is stable out of the influence area.

**Fig 9:**
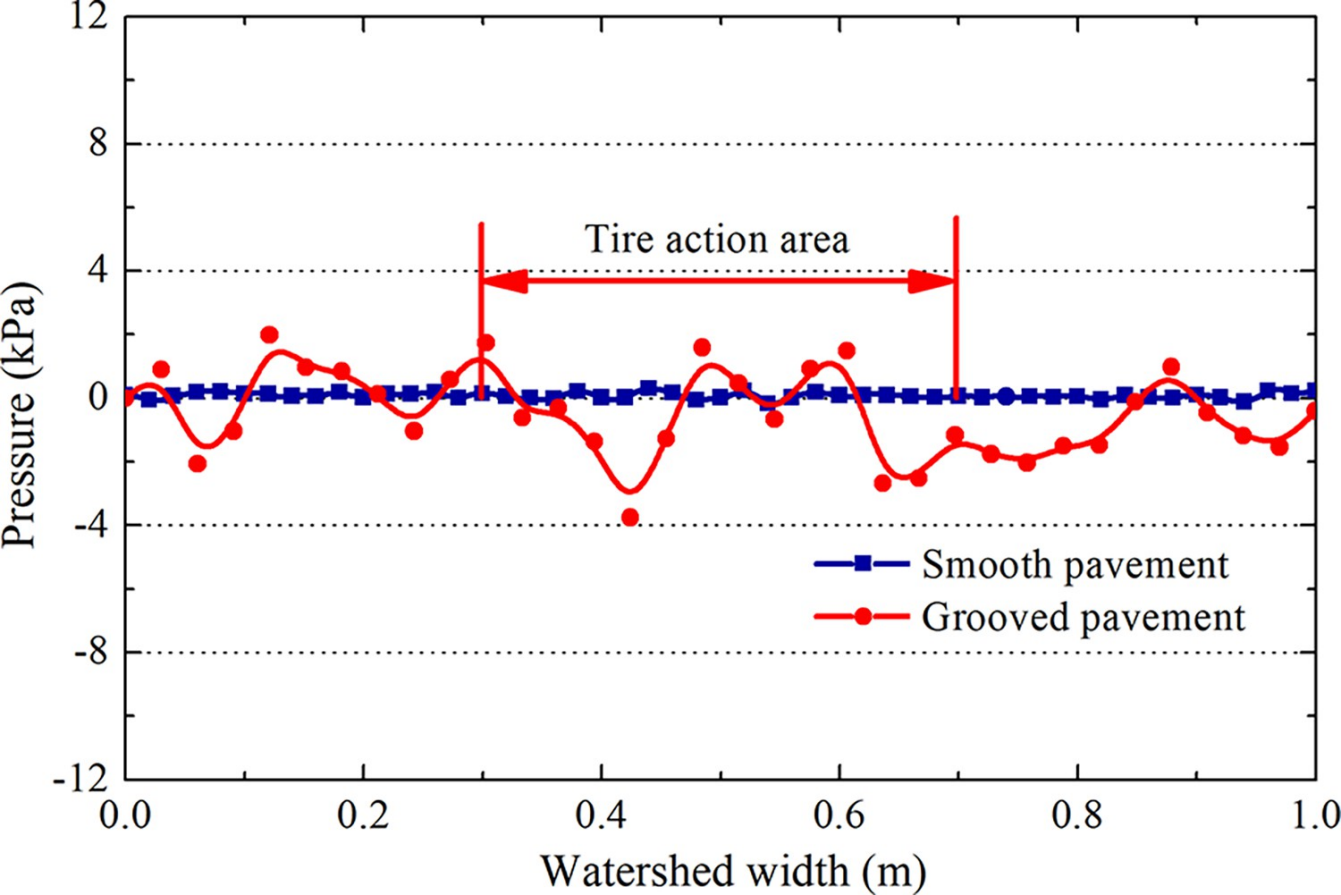
Hydrodynamic water pressure on the water surface of the A-A section.

**Fig 10:**
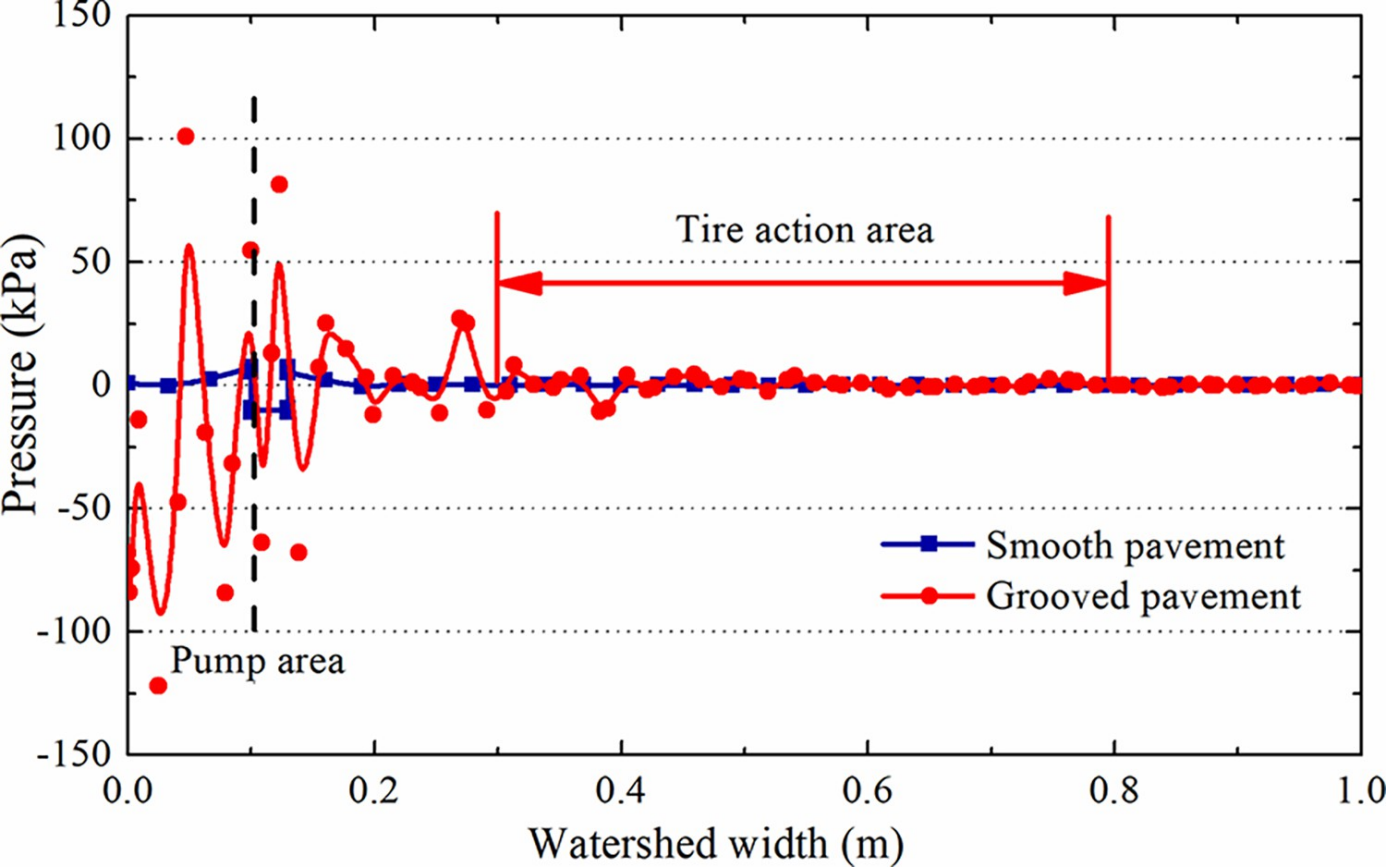
Hydrodynamic water pressure on the water.

Fig 9 shows that the pressure on the smooth pavement at section A-A is zero and that the water pressure on the grooved pavement fluctuates slightly, a maximum of only 3 kPa compared with the tire pressure of 1.14 MPa (the undulation in the flow field can be ignored). The pressure in the pump area, which is 0.1 m wide at the front of the catchment of section B-B, fluctuates wildly to a maximum of 90 kPa (Fig 10). The pressure of the tire action area after 0.3 m from the edge of the pump area is stable. Therefore, a catchment length of 1 m is sufficient to eliminate the influence of the boundary and the water pump area on the pressure.

**Fig 11:**
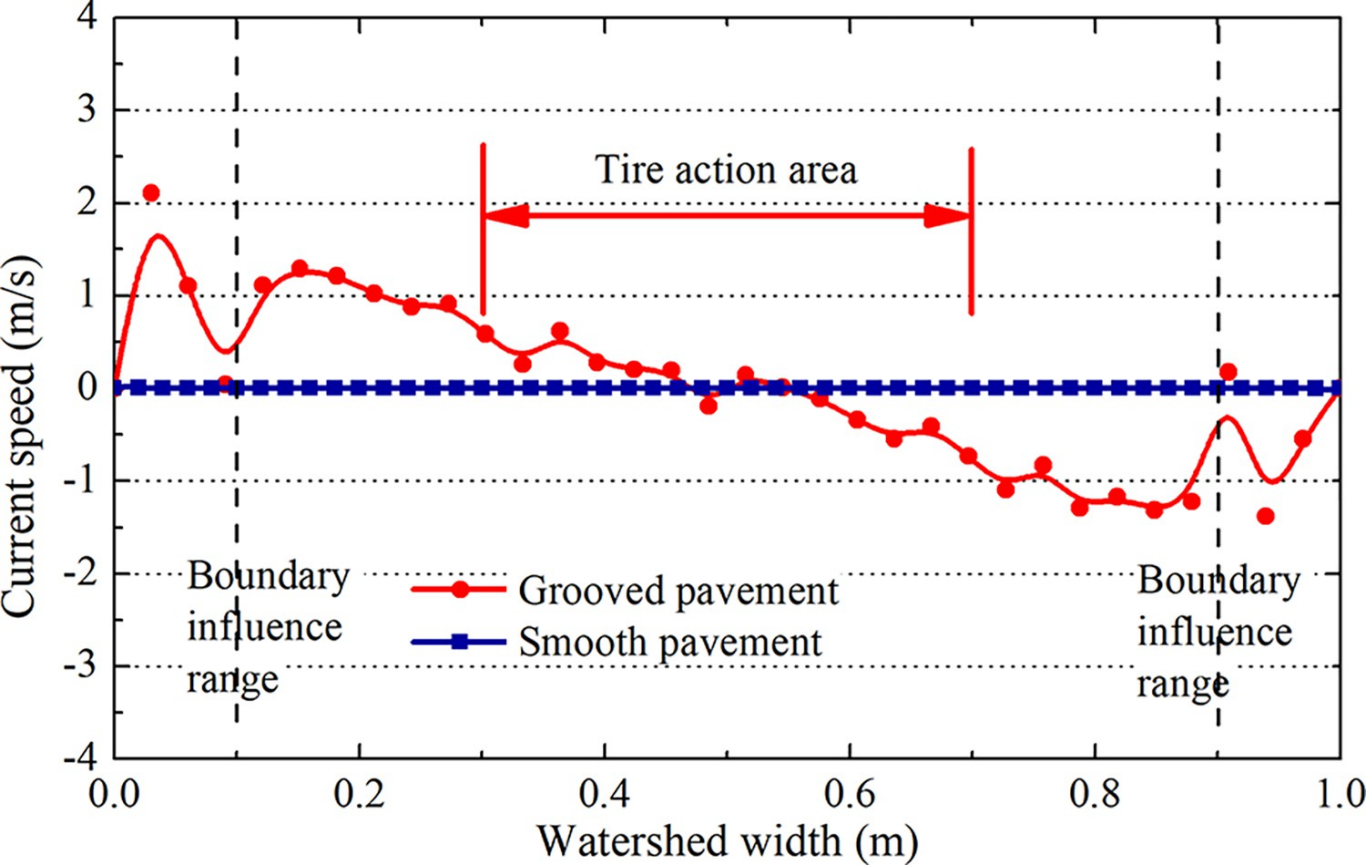
The flow velocity in the groove of the A-A section.

As shown in Fig 11, the extracted transverse flow velocity of the A-A section and the zero transverse flow velocity of the smooth pavement indicate that it does not possess transverse drainage capacity. In contrast, the transverse flow velocity of the grooved pavement gradually increases from the pavement’s center to both sides and in the opposite direction. This fully reflects the transverse drainage effect of grooves on the pavement and thus verifies the correctness of the grooved pavement hydrops model.

## Results and discussion

**Fig 12:**
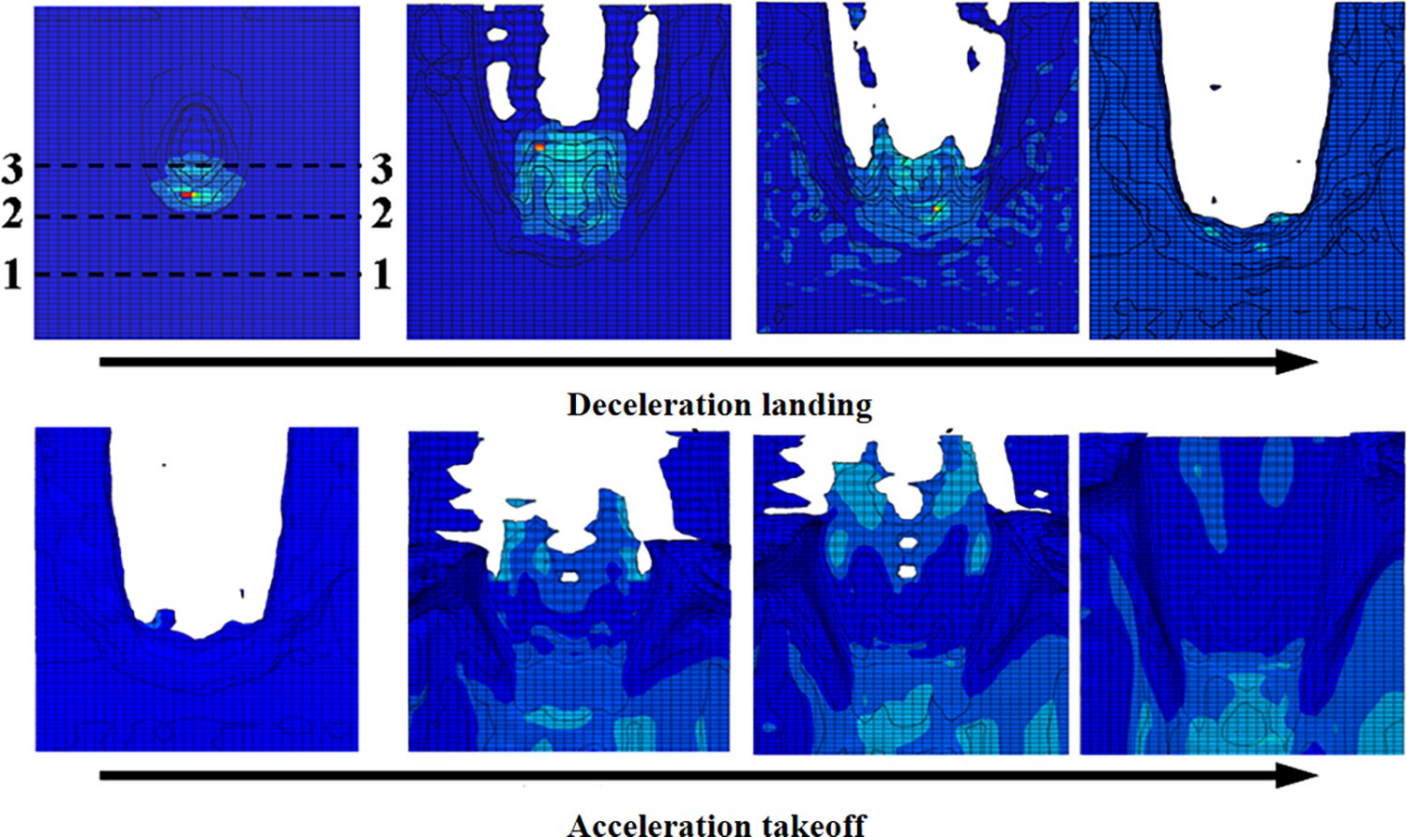
Diagram of the tire’s ground contact area.

**Fig 13:**
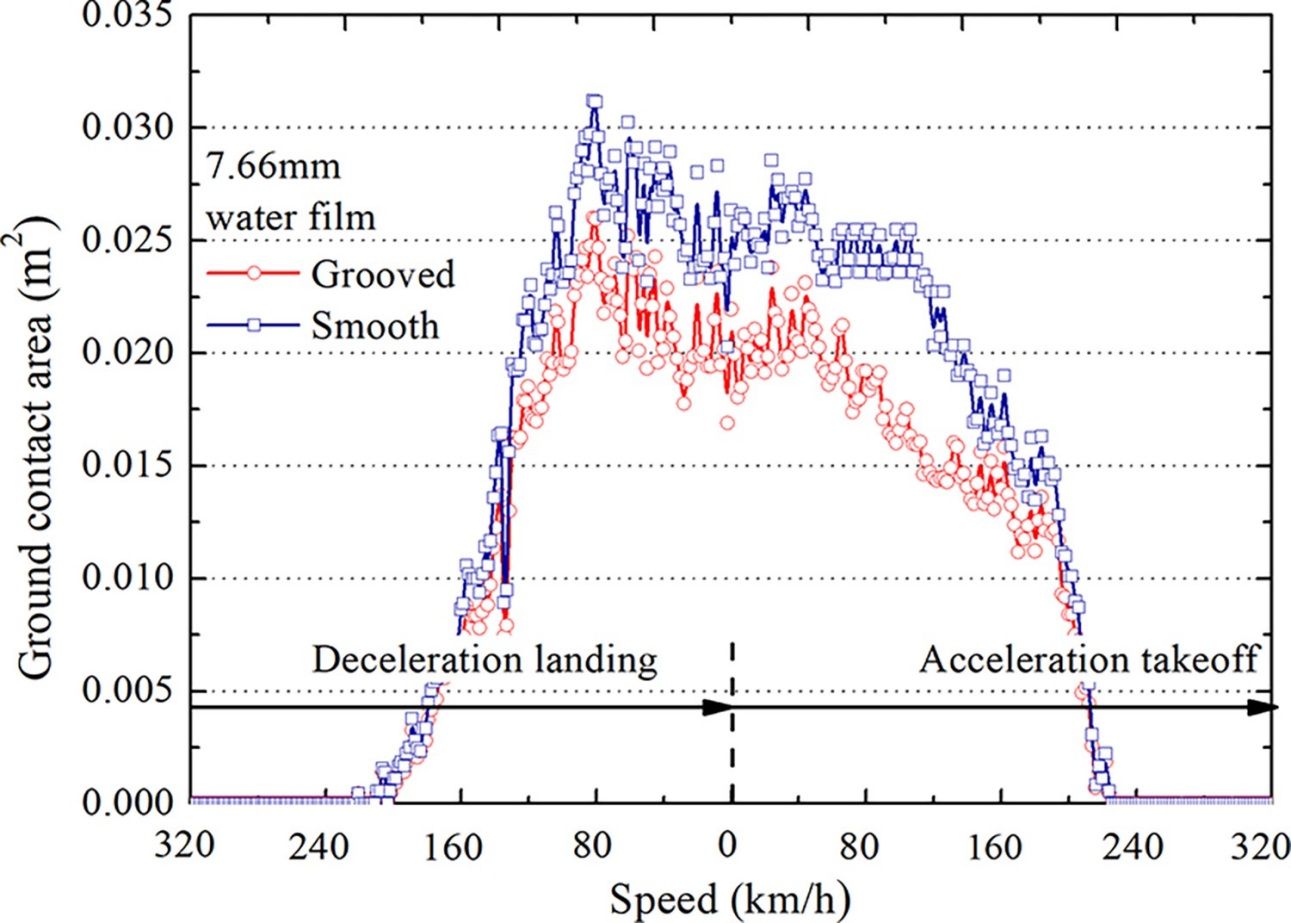
Curves of the tire’s ground contact area.

Applying deceleration and acceleration models to water flow simulates the tire-wet pavement interaction process during aircraft landing deceleration taxiing and takeoff acceleration taxiing [25–27]. The resulting change cloud diagrams and curves of the tire’s ground contact area are shown in Figs 12 and 13, respectively.

As shown in Fig 12, during deceleration landing, when the tire separates from the pavement, the contact area is zero; as the speed decreases, the tire starts to contact the ground, and the contact area gradually increases. During acceleration takeoff, the contact area changes in the opposite direction with increased speed. The contact area decreases gradually from the maximum until the tire is lifted off the ground. Fig 13 shows that the ground area of grooved pavement is smaller than that of smooth pavement at the same speed. When the rate is 0, the difference between the grooved and smooth pavement is 44 cm^2^. The maximum difference is 95 cm^2^, indicating that the interaction mechanism between the tire and grooved pavement differs from that of the smooth pavement.

### Analysis of the contact area of the tire and wet pavement

The NASA formula is a highly acceptable formula used to calculate the critical hydroplaning speed [28]. It assumes that the vertical load the tire bears when it reaches the actual hydroplaning state is balanced with the vertical support force *L*_*F*_ of the water film and that the tire is completely separated from the ground. The calculation formula is as follows:

$$L_{F}=C_{Lh}T_{F}=\frac{1}{2}C_{Lh}\rho v^{2}A$$ (3)

where *ρ* is the water film density (1000 kg/m^3^), *C*_*Lh*_ is the hydrodynamic pressure decomposition coefficient (0.7), and *A*_1_ is the contact area (m^2^) between the fluid and the tire.

The value of LF equals the aircraft’s single axle load:

$$P=\frac{L_{F}}{A_{2}}$$ (4)

where *P* is the tire pressure (kPa), *L*_F_ is the water film supporting force (kN) of the tire, and *A*_2_ is the contact area (m^2^) between the tire and the wet pavement.

Substituting Eq (4) into Eq (3) gives the following:

$$v_{h}=\sqrt{\frac{2PA_{2}}{\rho C_{Lh}A_{1}}}$$ (5)

where *v*_*h*_ is the critical hydroplaning speed (km/h).

**Fig 14:**
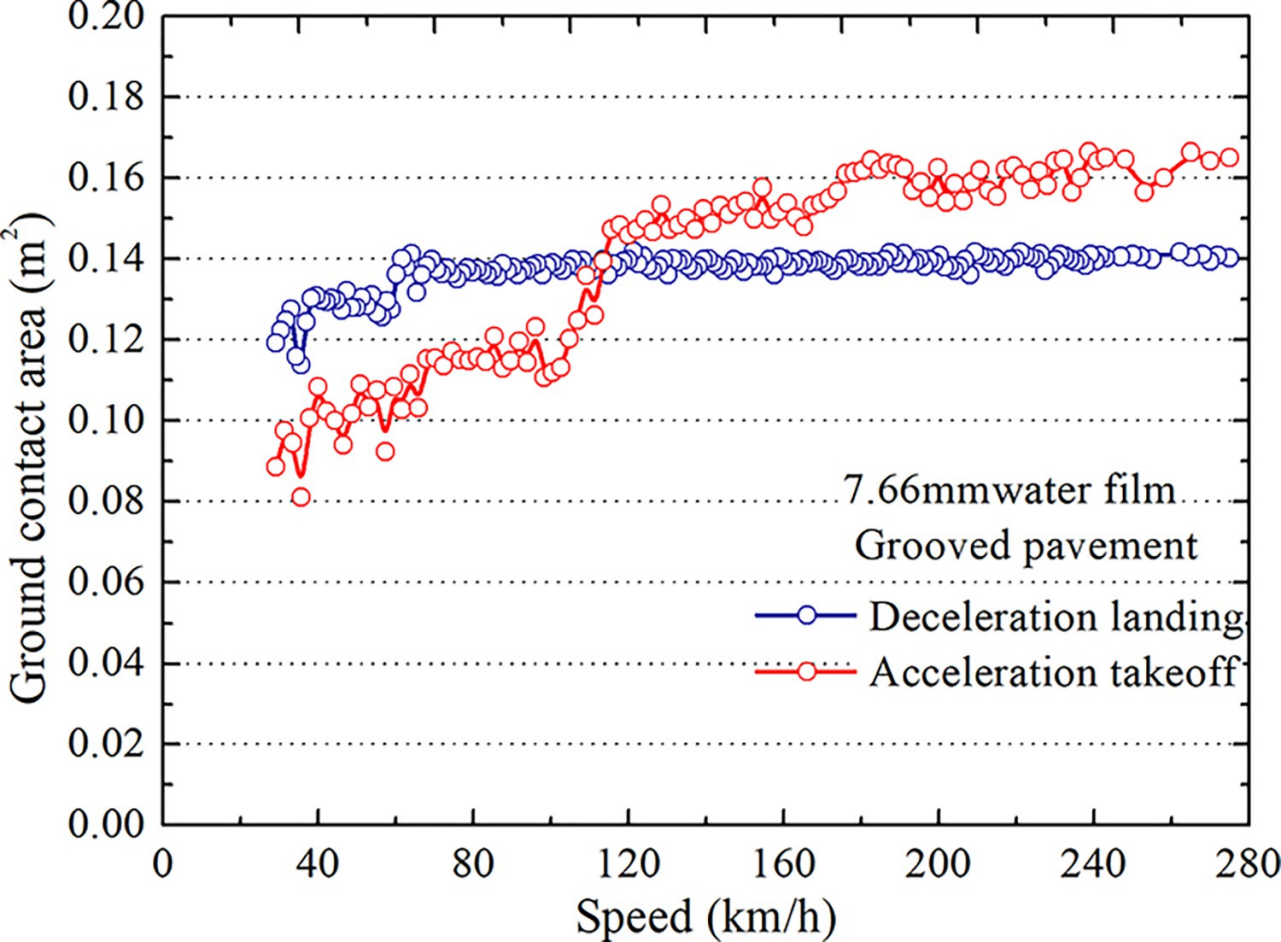
The contact area of the tire and grooved wet pavement.

**Fig 15:**
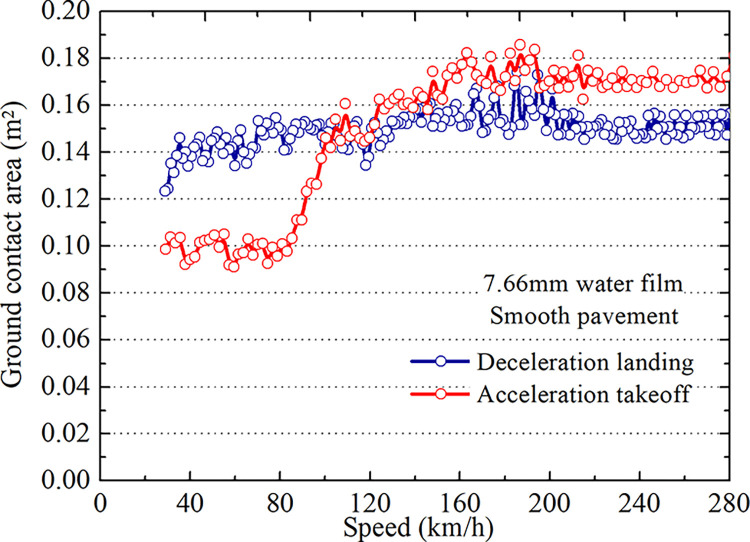
The contact area of the tire and smooth wet pavement.

Eq (5) shows that in wetting area *A*_1_, the critical hydroplaning conditions of the tire during takeoff and landing under the same pavement conditions and water film thicknesses are identical. The above model analyses the contact area *A*_2_ between the tire and the 7.66 mm ponding surface during takeoff and landing. The results are shown in Figs 14 and 15.

As seen in Figs 14 and 15, the contact area between the tire and pavement on the grooved pavement is smaller than that on the smooth pavement at the same speed, and the difference is 7.2%. The contact area *A*_2_ between the tire and wet pavement on smooth and grooved surfaces changes with the taxiing speed and differs during landing and takeoff. When the taxiing speed is less than 120 km/h, the contact area during landing is more significant than that during takeoff. When the taxiing speed is greater than 120 km/h, the contact area *A*_2_ during the takeoff is more prominent than during landing. The speed range is the hydroplaning range. From Eq (5), it can be concluded that the critical hydroplaning speed for takeoff differs from that for landing, and the former is higher than the latter.

### Analysis of the hydrodynamic pressure of the tire-water film

**Fig 16:**
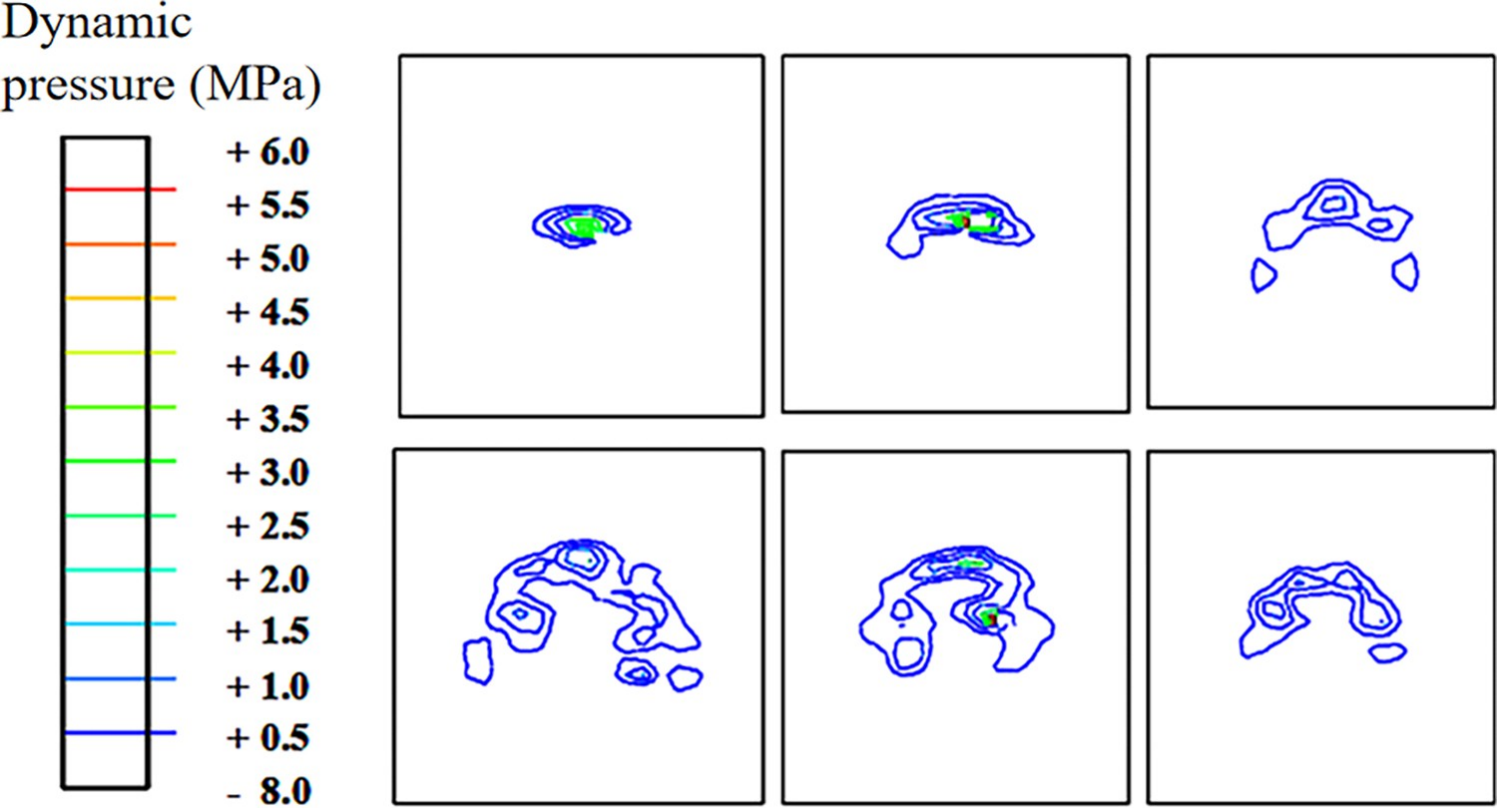
Diagram of the hydrodynamic pressure on the grooved pavement during aircraft landing.

**Fig 17:**
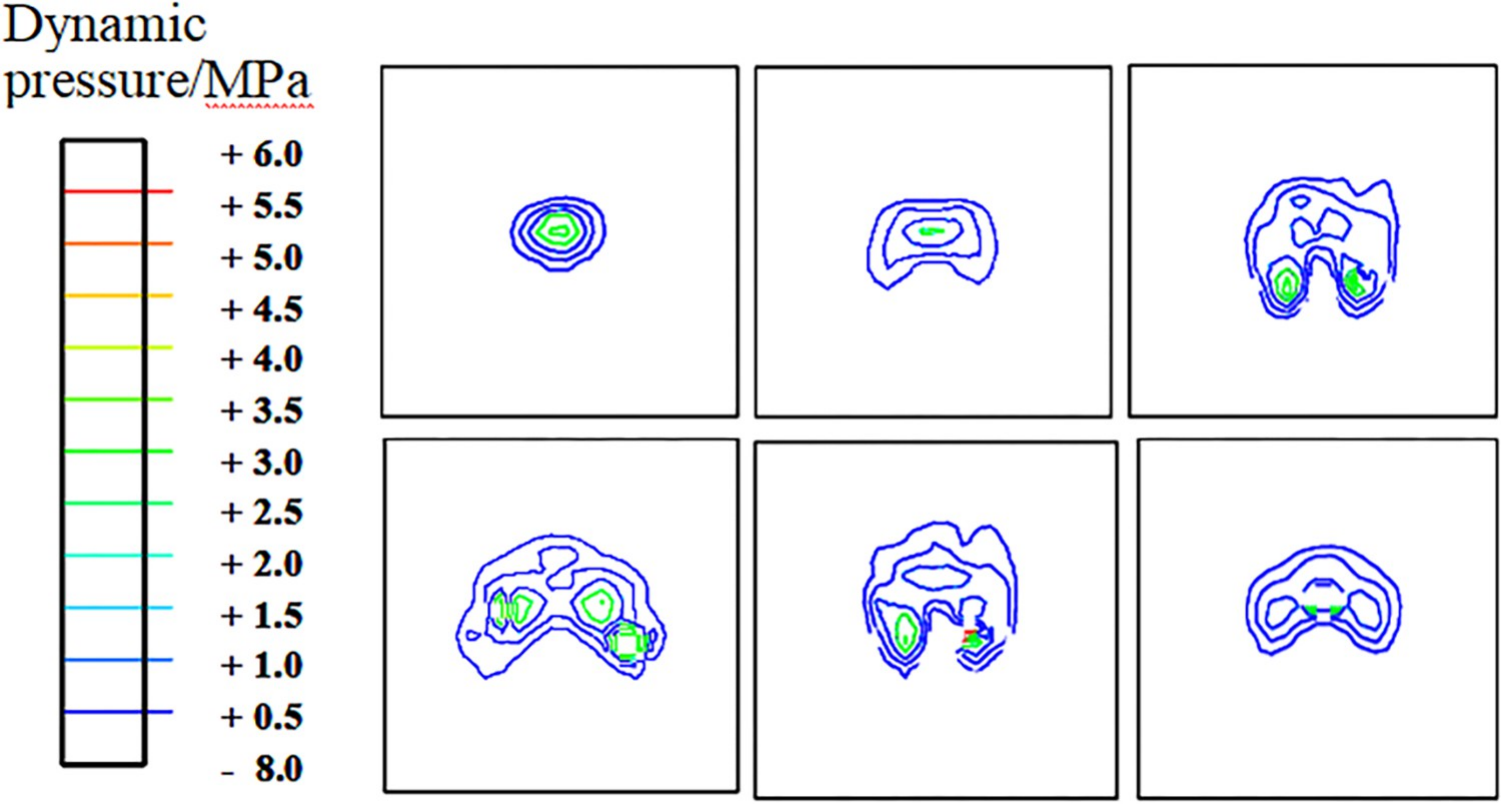
Diagram of the hydrodynamic pressure on the smooth pavement during aircraft landing.

In this section, the variation process of hydrodynamic pressure during landing and takeoff is analyzed. Here, only the pressure distribution in the watershed during landing is provided. Figs 16 and 17 show that the pressure of the water film in the contact area is high at the instant of landing. After the tire decelerates, the squeezing action of the water film decreases, and the pressure in the watershed decreases gradually. However, the force of the tire’s leading edge is still high. Comparatively speaking, the water film pressure of the tire landing on smooth pavement is higher than that on grooved pavement and has a more extensive influence range.

**Fig 18:**
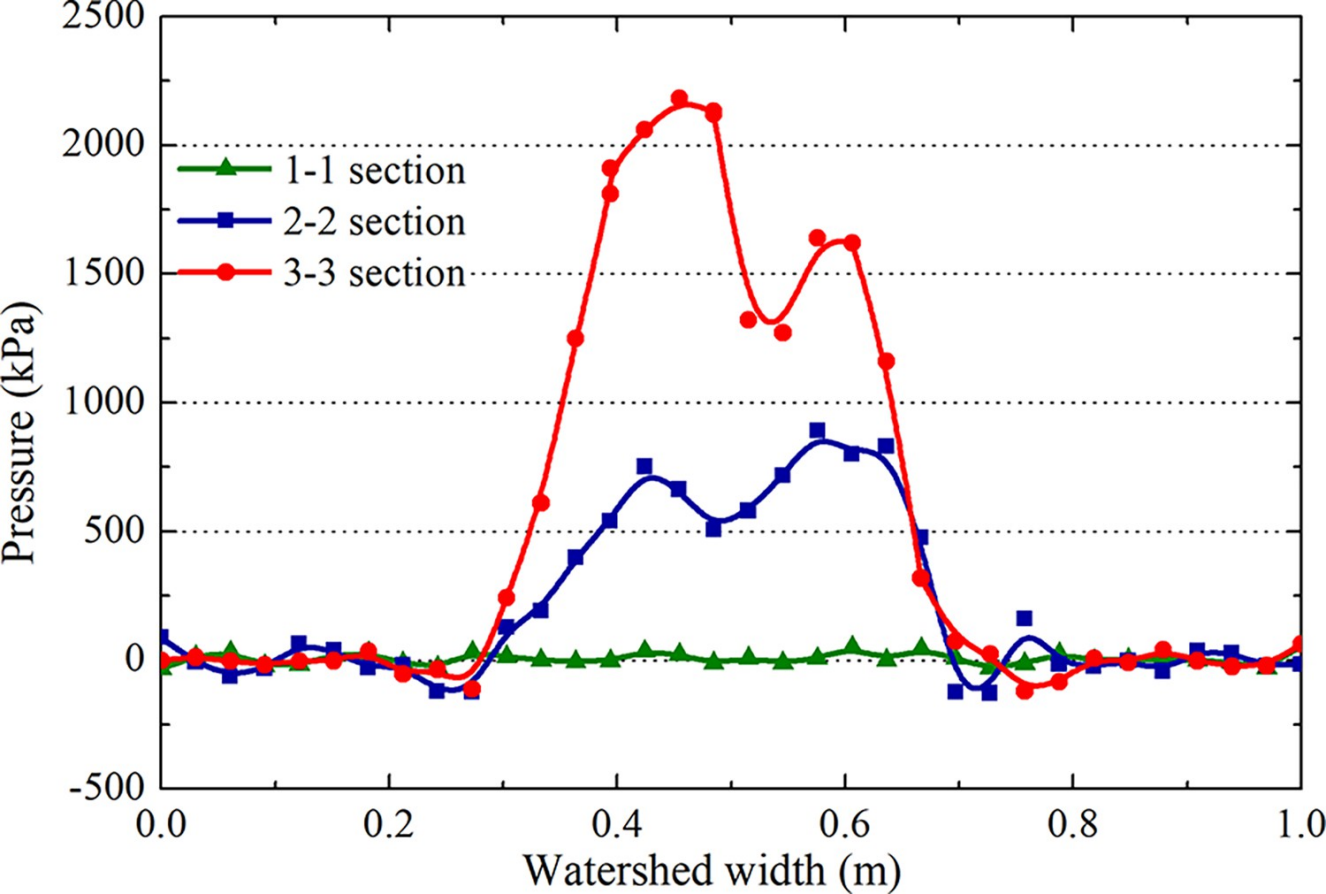
Hydrodynamic pressure during the deceleration landing stage (grooved wet pavement).

**Fig 19:**
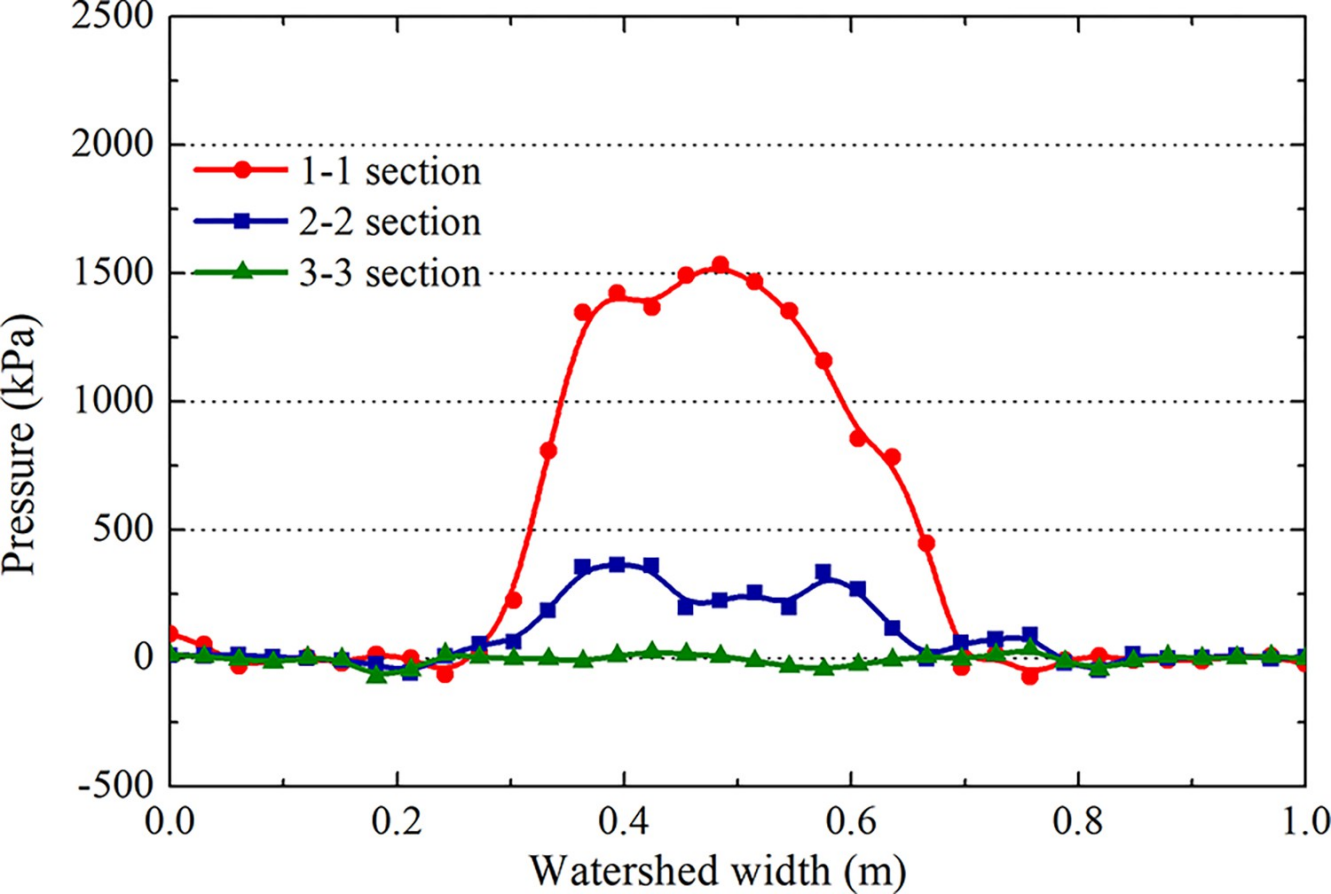
Hydrodynamic pressure during the acceleration takeoff stage (grooved wet pavement).

The pressure distributions of the water film layer in sections 1–1, 2–2, and 3–3 of Fig 12 are analyzed during the landing and takeoff phases on the grooved and smooth pavements at the same speed (Figs 18 and 19). The hydrodynamic pressure in the tire area is significantly higher than that in the edge area. The force of section 3–3 is much higher than that of sections 2–2 and 1–1, and the peak hydrodynamic pressure of section 3–3 generated by the deceleration landing stage is 2.2 MPa, which is 1.5 MPa higher than that of the acceleration takeoff stage, with a difference of 46.6%. It can be concluded that the critical hydroplaning speed in the deceleration landing stage is lower than that in the takeoff stage; hydroplaning is more likely to occur in the landing stage.

**Fig 20:**
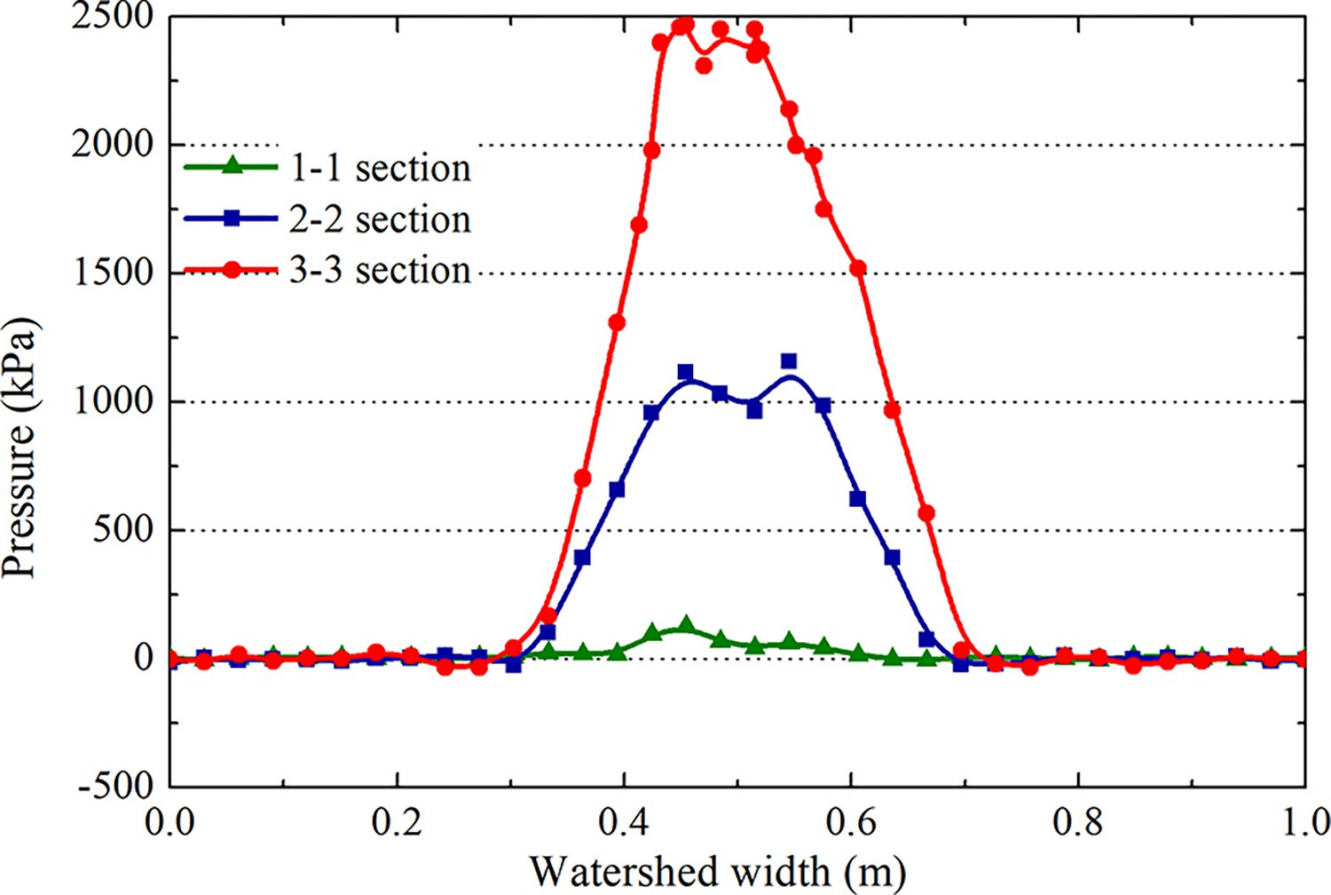
Hydrodynamic pressure during the deceleration landing stage (smooth wet pavement).

**Fig 21:**
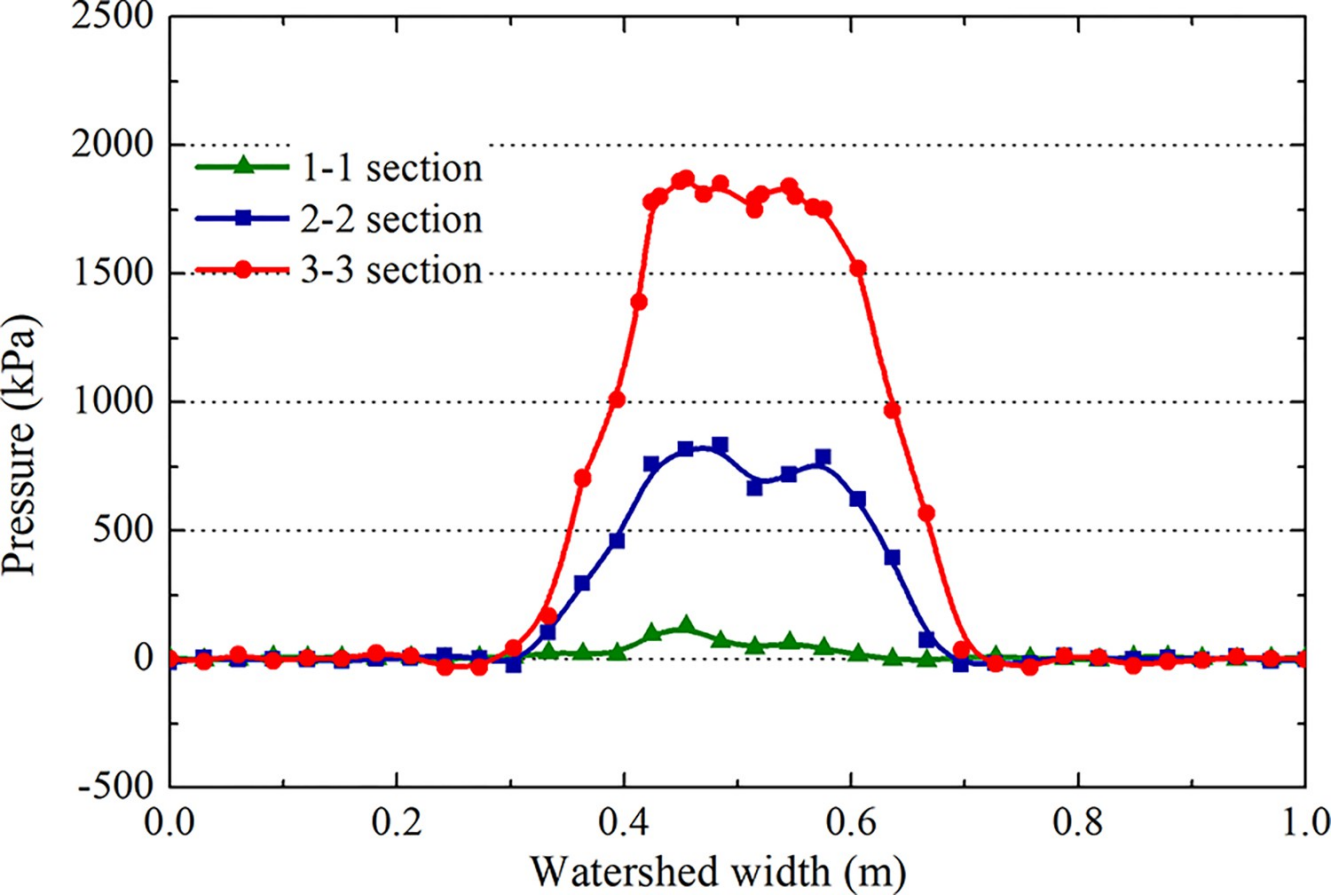
Hydrodynamic pressure during the acceleration takeoff stage (smooth wet pavement).

Comparing the above results (Figs 18–21), the distribution law of the hydrodynamic pressure on smooth pavement is the same as that on grooved pavement. However, the hydrodynamic force on each section of smooth pavement is 42% higher than that on grooved pavement. The peak pressure of section 3–3 on accelerated takeoff is 2.5 MPa, upwards of 1.8 MPa on decelerated landing, with a difference of 38.9%. Similarly, the critical hydroplaning speed of decelerated landing is lower than that of accelerated takeoff.

### Analysis of pavement support force and tire lifting force

**Fig 22:**
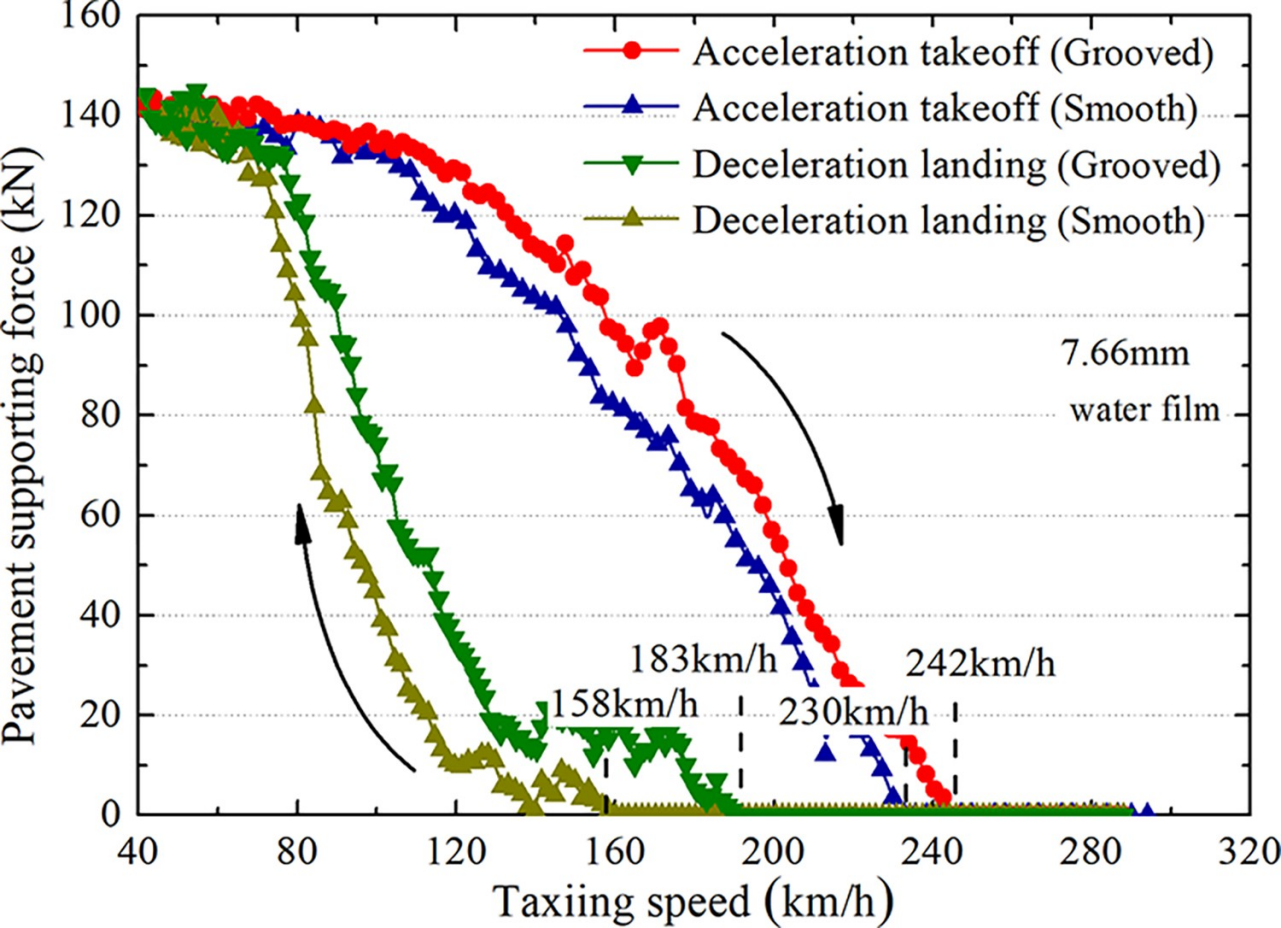
Support force of the wet pavement (7.66 mm water film).

The tire support force on smooth and grooved surfaces is extracted to analyze the variation rule of the tread support force with taxiing speed and to obtain the point [29] at which the tread support force tends to zero. The corresponding taxiing speed is the critical hydroplaning speed under this condition (Fig 22). At the same rate, the tire support force of the groove pavement is greater than that of the smooth pavement, with a difference of approximately 20 kN. The critical hydroplaning speed in the groove pavement is greater than that in the smooth pavement, with a difference of approximately 20 km/h. There are laws that the critical hydroplaning speed for deceleration landing is lower than that for accelerated takeoff on smooth and grooved surfaces. The specific values are shown in Table 3.

**Table 3:** Hydroplaning speeds on smooth and grooved pavements (7.66 mm water film).

	Smooth (A) km/h	Grooved (B) km/h	Promotion rate % (B-A)/A
Deceleration landing stage	158	183	15.8
Acceleration takeoff stage	230	242	5.2
Difference (km/h)	72	59	/

Fig 22 and Table 3 show that both the smooth and grooved pavements have upper and lower critical hydroplaning speeds under the condition of 7.66 mm wet pavement. The critical hydroplaning speed for accelerated takeoff is approximately 60 km/h higher than that for decelerated landing. At the same time, the critical hydroplaning speed of the grooved pavement is higher than that of the smooth pavement, 15.8% and 5.2% higher than that during landing and takeoff, respectively. Therefore, it is necessary to consider the influence of pavement texture on critical hydroplaning speed. To further prove the occurrence of hydroplaning, the resultant forces of pavement and water film on the tire are extracted and analyzed (Table 4). The resultant tire force corresponding to the critical hydroplaning speed is greater than the axle load (153 kN) when the pavement tire support force is 0, and the resultant tire force is greater than the axle load; the pavement is separated from the tire and undergoes hydroplaning.

**Table 4:** Total force on the tire (hydroplaning moment).

Pavement condition	Smooth pavement		Grooved pavement	
Taxiing state	Landing	Takeoff	Landing	Takeoff
Tire resultant (kN)	155.4	153.6	156.8	154.3

### The upper and lower bounds of the critical hydroplaning speed

Based on the results of the contact area analysis, the hydrodynamic pressure analysis, and the pavement support force analysis of the tire-wet pavement interaction mentioned above, it is proposed that the critical hydroplaning speed has upper and lower limits; that is, there is a critical hydroplaning speed for takeoff and a critical hydroplaning speed for landing.

The regression analysis of substantial test data [2] suggests that *C*_*Lh*_ is equal to 0.7. Taking it into Eq (5), the NASA critical hydroplaning speed formula is obtained:

$$\nu_{h}=6.36\sqrt{P}$$ (6)

According to the NASA formula and the research results in this paper, the following upper and lower limit formulas for critical hydroplaning speed are proposed:

Upper limit critical hydroplaning speed:

$$v_{up}=6.36\alpha \sqrt{P_{}}$$ (7)

Lower critical hydroplaning speed:

$$v_{low}=6.36\beta {\sqrt{P}}_{}$$ (8)

Here, *α* and *β* are the comprehensive correction coefficients related to the thickness of stagnant water and the type of machine. The various influencing factors *α* and *β* are as follows:

$$\alpha =\prod_{i=1}^{n}\alpha_{i}$$ (9)

$$\beta =\prod_{i=1}^{n}\beta_{i}$$ (10)

### Analysis of the critical hydroplaning speed for multiple models

**Fig 23:**
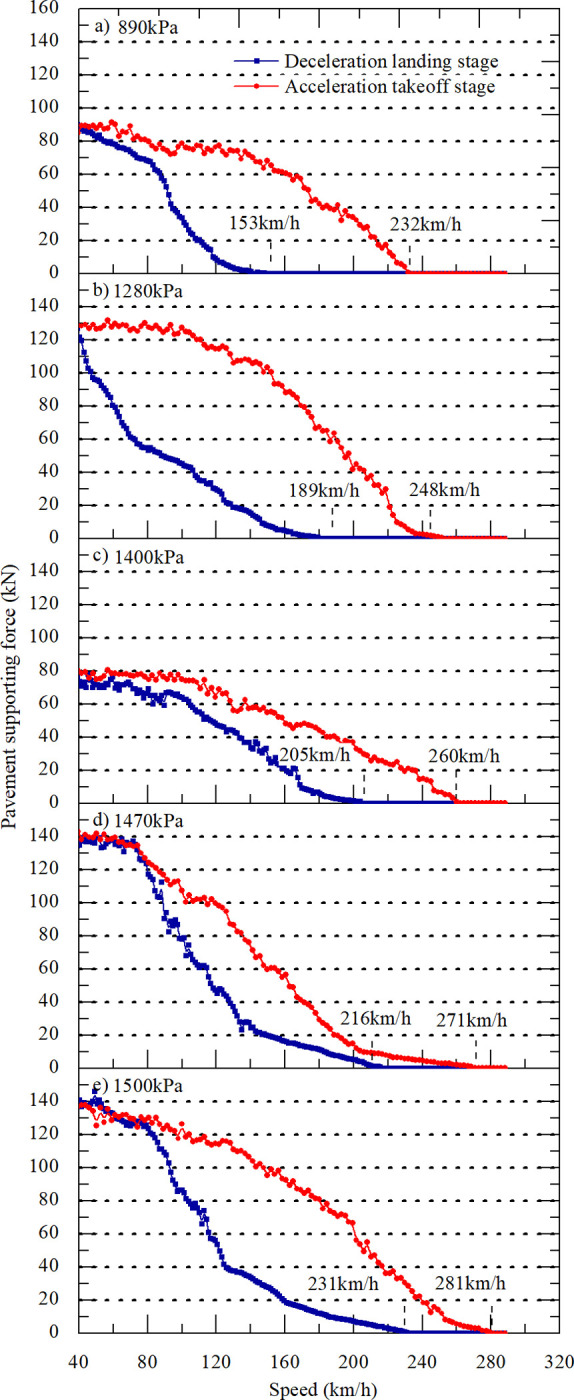
Support force of wet pavement for different aircraft.

**Fig 24:**
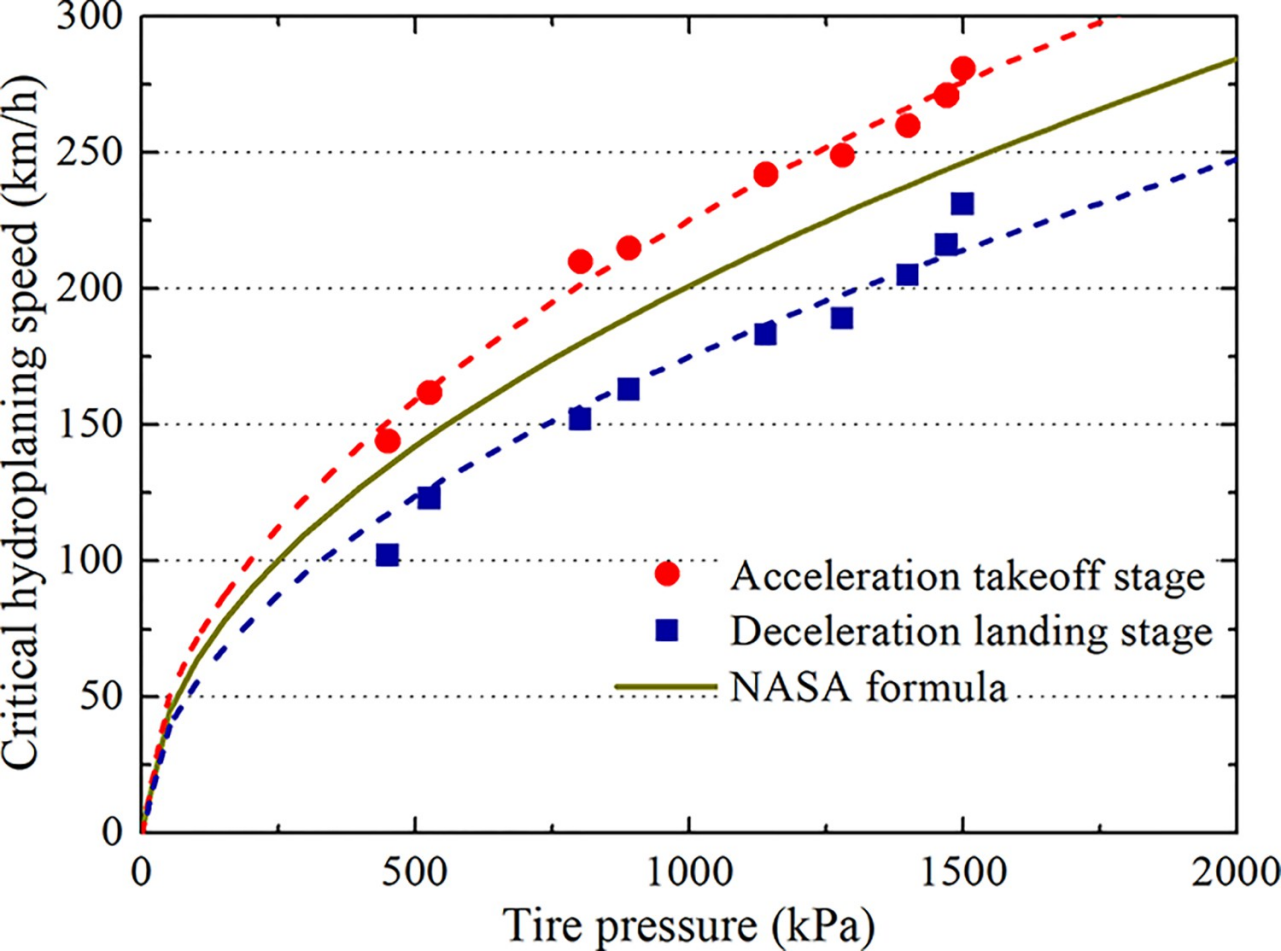
Relationship between tire pressure and critical hydroplaning speed.

Five different aircraft tire pressures are selected according to the mainstream domestic types, and the operating conditions of the 7.66 mm water film are analyzed (Table 5). Simulation analysis of accelerated takeoff and decelerated landing for each type on the grooved pavement is carried out, resulting in variable curves that represent the pavement support force. Thus, the critical hydroplaning speeds of different aircraft can be obtained, as shown in Fig 23 and Table 6. Curve fittings of the upper and lower limits of the critical hydroplaning speeds at each tire pressure are shown in Fig 24. Additionally, these results are compared with the predicted values obtained from the NASA formula.

**Table 5:** Aircraft types.

Type	Single wheel axle load (kN)	Tire pressure (kPa)
A319	141.8	890
A320	153.2	1140
B777-200	189.0	1280
B737-300	100.8	1400
A380	183.4	1470
B777-200LR	174.4	1500

**Table 6:** Hydroplaning speeds of different aircraft (km/h).

Tire pressure (kPa)	450	526	801	890	1140	1280	1400	1470	1500
*v*_*up*_	144	162	210	232	242	248	260	271	281
*v*_*low*_	102	123	152	153	183	189	205	216	231
Difference	42	39	58	79	59	59	55	55	50

Fig 24 shows that the upper and lower critical hydroplaning speeds increase with increasing tire pressure. During the deceleration landing stage, the critical hydroplaning speed is lower than the predicted value obtained from the NASA formula. If the NASA formula is used to calculate the landing speed, then hydroplaning accidents are likely to occur. During the acceleration takeoff stage, the critical hydroplaning speed is higher than that predicted by the NASA formula. By fitting the critical hydroplaning speed under each tire pressure and forecasting the hydroplaning pace when landing on wet pavement, the coefficient of correction of the hydroplaning rate on grooved pavement needs to be reduced 13% when applying the NASA formula. Alternatively, the NASA formula can be safely used to predict the hydroplaning rate at takeoff.

### Analysis of the critical hydroplaning speed with different water film thicknesses

**Fig 25:**
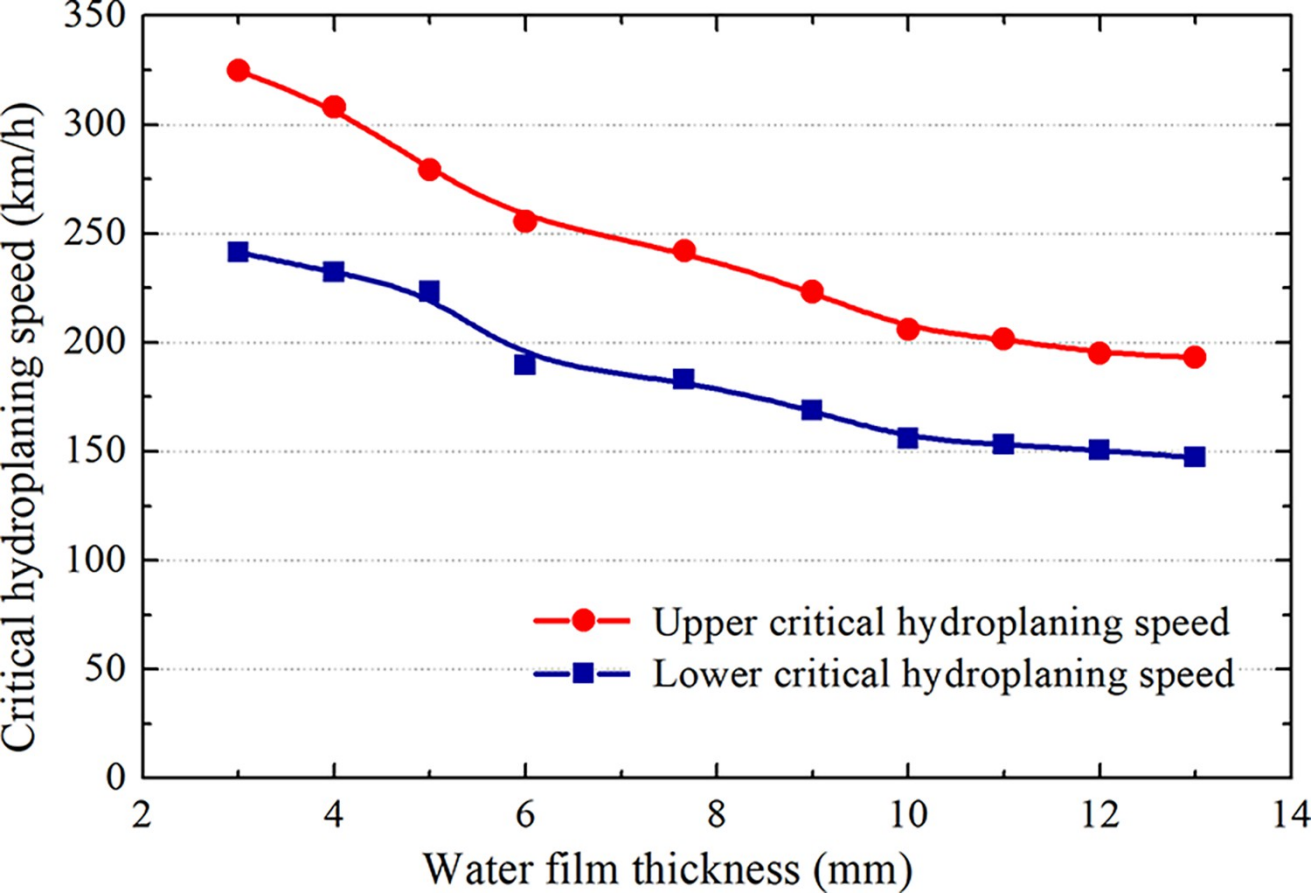
Hydroplaning speeds at different water film thicknesses.

The NASA formula is based on 7.66 mm water film thickness test conditions. Notably, water film thicknesses up to 13 mm are allowed for takeoff and landing according to the Regulation Standards for Operating Management of Wet and Polluted Runways for Air Carriers (AC-121-FS-2009-33). Therefore, the thickness of the water film is within 13 mm. Under a standard tire pressure of 1140 kPa, the water film thickness on the grooved pavement is changed to analyze the critical hydroplaning speed, as shown in Fig 25 and Table 7.

**Table 7:** Hydroplaning speeds of different water film thicknesses (km/h).

Water film	3 mm	4 mm	5 mm	6 mm	7.66 mm	9 mm	10 mm	11 mm	12 mm	13 mm
*α*_2_	1.34	1.27	1.15	1.06	1.00	0.92	0.85	0.83	0.81	0.80
*β*_2_	1.32	1.27	1.22	1.04	1.00	0.92	0.85	0.84	0.82	0.80

Fig 25 shows that the critical hydroplaning speed decreases with increasing water film thickness. When the water film thickness is less than 7.66 mm, the critical hydroplaning rate is more significant (NASA test condition). When the water film thickness is 3 mm, the critical hydroplaning speed is approximately 33% higher than that at 7.66 mm. However, when the water film thickness is greater than 7.66 mm, the critical hydroplaning speed decreases, especially when the water film thickness reaches 13 mm, and the hydroplaning speed is approximately 20% lower than that at 7.66 mm.

The upper critical hydroplaning speed correction coefficient of the water film thickness is defined as *α*_2_. The value of this coefficient is the ratio of the upper critical hydroplaning speed corresponding to the water film thickness and the upper critical hydroplaning speed corresponding to a water film thickness of 7.66 mm. The expression is as follows:

α2=Vup(h)Vup(7.66mm) (11)

The correction coefficient for the lower critical hydroplaning speed concerning the water film thickness is *β*_2_. Its definition is the same as that for *α*_2_, and the expression is as follows:

β2=Vlow(h)Vlow(7.66mm) (12)

**Fig 26:**
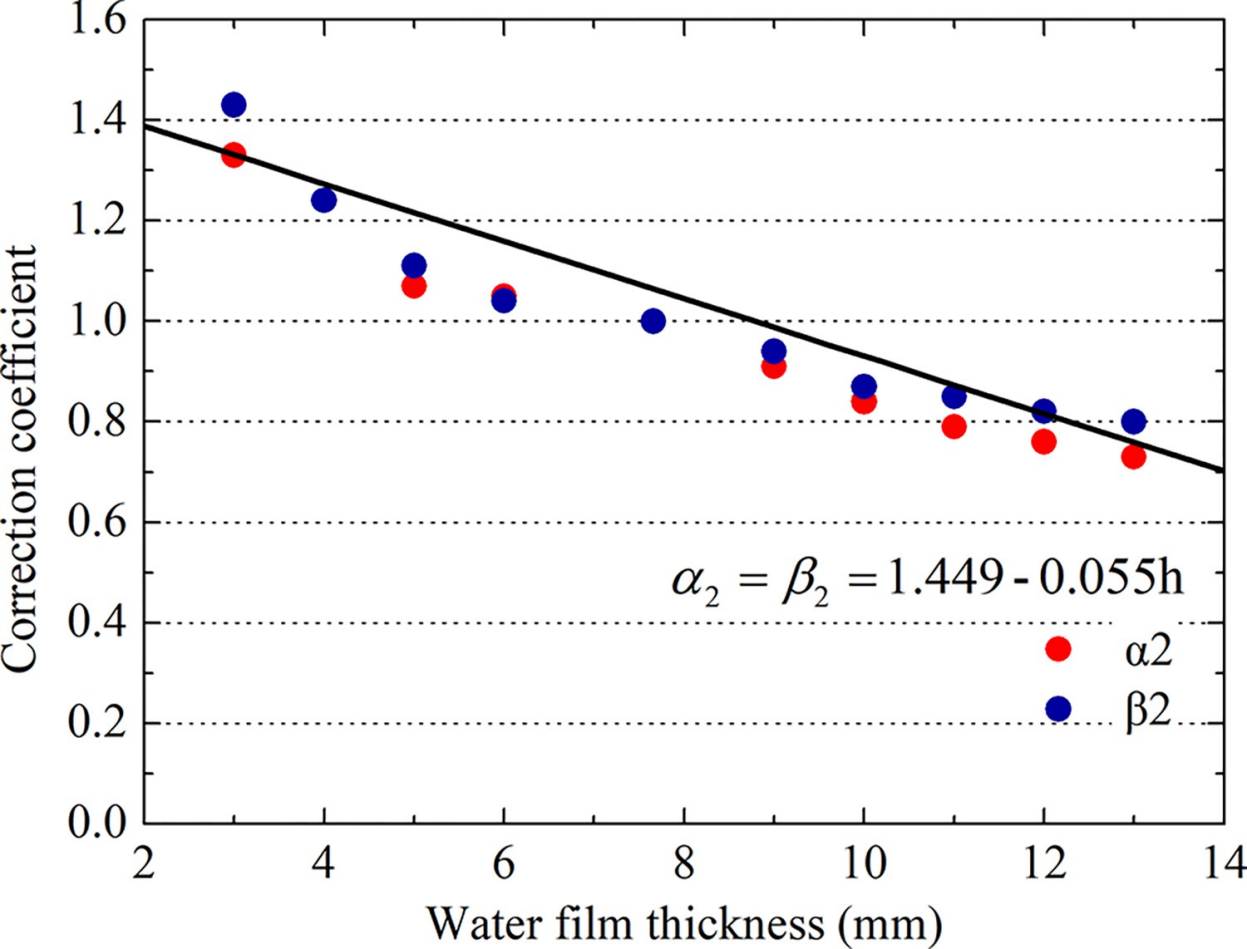
Correction coefficient of the water film thickness.

As shown in Fig 26, the water film thickness correction coefficient decreases with increasing water film thickness. When the water film thickness is less than 7.66 mm, the correction coefficient of the water film thickness is greater than 1. Thus, it is safe to employ the NASA formula to predict the critical sliding water speed. When the water film thickness is greater than 7.66 mm, the correction coefficient of the water film thickness is less than 1. Then, the aircraft may experience hydroplaning if the NASA formula is used to predict the critical hydroplaning speed.

As shown in Fig 26, *α*_2_ and *β*_2_ are almost identical, so the linear fitting formula is as follows:

$$\alpha_{2}=\beta_{2}=1.4490‐0.055h$$ (13)

## Conclusions

1. Comparison of grooved and smooth wet pavements. The aircraft tire ground contact area gradually increases during deceleration landing but gradually decreases to 0 during acceleration takeoff. Moreover, the contact area is lower on grooved pavement than on smooth pavement by 7.2%. The hydrodynamic pressure at the tire leading edge is lower on grooved pavement than on smooth pavement, by 37.2%.
2. During the acceleration takeoff stage, the tire ground contact area is higher than that during deceleration landing, and the hydrodynamic pressure produced by acceleration takeoff is lower than that of decelerated landing in the speed range of hydroplaning. Therefore, there are upper and lower limit critical hydroplaning velocities between the different taxiing states, and the difference is approximately 60 km/h.
3. The hydroplaning behavior of aircraft with different tire pressures is analyzed. The correction factor of the critical hydroplaning speed on the grooved pavement exhibits an upper limit of 1.12 and a lower limit of 0.87. The upper and lower limits of the correction factor of the critical hydroplaning speed for a water film on the grooved pavement have the following rules. When the water film thickness is greater than 7.66 mm, the correction factor is in the range of 0.8~1, and when the water film thickness is less than 7.66 mm, the correction factor is in the range of 1~1.32. Landing taxiing is dangerous when the NASA formula is used to predict the critical hydroplaning speed. The prediction must be reduced by 13%, which is considered to be safe during takeoff taxiing.
